# Toughening Effect of 2,5-Furandicaboxylate Polyesters on Polylactide-Based Renewable Fibers

**DOI:** 10.3390/molecules28124811

**Published:** 2023-06-16

**Authors:** Giulia Fredi, Edoardo Zonta, Alessandro Dussin, Dimitrios N. Bikiaris, George Z. Papageorgiou, Luca Fambri, Andrea Dorigato

**Affiliations:** 1 Department of Industrial Engineering and INSTM Research Unit, University of Trento, Via Sommarive 9, 38123 Trento, Italy; edoardo.zonta@unitn.it (E.Z.); luca.fambri@unitn.it (L.F.); andrea.dorigato@unitn.it (A.D.); 2 Laboratory of Polymer Chemistry and Technology, Chemistry Department, Aristotle University of Thessaloniki, 54124 Thessaloniki, Greece; dbic@chem.auth.gr; 3 Department of Chemistry, University of Ioannina, 45110 Ioannina, Greece; gzpap@uoi.gr

**Keywords:** fibers, polylactide, poly(alkylene furanoate)s, furan polyesters, blends, compatibilization, renewable polymers

## Abstract

This work presents the successful preparation and characterization of polylactide/poly(propylene 2,5-furandicarboxylate) (PLA/PPF) and polylactide/poly(butylene 2,5-furandicarboxylate) (PLA/PBF) blends in form of bulk and fiber samples and investigates the influence of poly(alkylene furanoate) (PAF) concentration (0 to 20 wt%) and compatibilization on the physical, thermal, and mechanical properties. Both blend types, although immiscible, are successfully compatibilized by Joncryl (J), which improves the interfacial adhesion and reduces the size of PPF and PBF domains. Mechanical tests on bulk samples show that only PBF is able to effectively toughen PLA, as PLA/PBF blends with 5–10 wt% PBF showed a distinct yield point, remarkable necking propagation, and increased strain at break (up to 55%), while PPF did not show significant plasticizing effects. The toughening ability of PBF is attributed to its lower glass transition temperature and greater toughness than PPF. For fiber samples, increasing the PPF and PBF amount improves the elastic modulus and mechanical strength, particularly for PBF-containing fibers collected at higher take-up speeds. Remarkably, in fiber samples, plasticizing effects are observed for both PPF and PBF, with significantly higher strain at break values compared to neat PLA (up to 455%), likely due to a further microstructural homogenization, enhanced compatibility, and load transfer between PLA and PAF phases following the fiber spinning process. SEM analysis confirms the deformation of PPF domains, which is probably due to a “plastic–rubber” transition during tensile testing. The orientation and possible crystallization of PPF and PBF domains contribute to increased tensile strength and elastic modulus. This work showcases the potential of PPF and PBF in tailoring the thermo-mechanical properties of PLA in both bulk and fiber forms, expanding their applications in the packaging and textile industry.

## 1. Introduction

Being biodegradable and/or derived from renewable resources, bioplastics can represent a more sustainable alternative to conventional plastics [1]. This is especially true in all those applications where the exceptional versatility and unmatched physical properties of traditional polymers are overshadowed by their petrochemical origin and challenging waste management [2]. In fact, the carbon footprint reduction at the early life cycle stages and the alternative routes for waste disposal provided by bioplastics make them a credible and important ally in the pathway toward a more sustainable society [3,4]. Notwithstanding this, the market for bioplastics currently represents less than 1% of the total global plastic production market [5], due to their generally higher price and/or poorer performance compared to conventional polymers. Hence, considerable research and development endeavor is still needed to find economically viable routes for bioplastics’ production, to enhance their physical-mechanical properties, and to tailor their biodegradation rate. Only in this way, the economic and environmental sustainability of bioplastics will be full [6].

Among the applications in which biopolymers can thrive is that of sustainable synthetic textile fibers. For instance, fully bioderived and biodegradable polymers, such as poly(lactic acid) (PLA), have a promising future in the production of fibers in disposable garments and home textiles [7]. PLA exhibits acceptable properties for these applications, such as an elastic modulus of approx. 3–4 GPa and a tensile strength of approx. 50–70 MPa, and can be easily processed using conventional manufacturing equipment, also given its lower melting temperature (T_m_ = 170 °C) compared to that of other typical textile polymers such as poly(ethylene terephthalate) (PET) (T_m_ = 260 °C) [8,9,10,11]. PLA filaments can be manufactured through various spinning methods, with melt spinning being the most commonly used [12,13]. Despite these important advantages, PLA suffers from some severe drawbacks in the processing phase, such as poor melt strength, narrow processing window, degradation, and poor dimensional stability [14,15], which may cause deformations, cracking, unacceptable fiber roughness, and reduction in molecular weight. Additionally, PLA’s intrinsic brittleness and poor strain at break (<10%) limit its widespread applications in many packaging and textile fields [16]. These substantial shortcomings have been generally addressed via additives, such as plasticizers and chain extenders [17,18], and/or through physical blending with other traditional and bioderived polymers [19,20]. Accordingly, despite the recent advancements in blending and copolymerizing PLA with other bioderived compounds [21], the quest for an appropriate, easily accessible, and cost-effective bio-additive that enhances PLA’s ductility while preserving its stiffness and strength persists as an ongoing research challenge.

An attractive class of biopolymers that can be blended with PLA is that of the poly(alkylene furanoate)s (PAFs). These polymers embody a viable biobased alternative to petrochemical-derived terephthalate polyesters and are synthesized from furan-2,5-dicarboxylic acid (FDCA), which has been listed among the top 12 green platform chemicals obtained from sugar fermentation [22]. PAFs have been synthesized via the polycondensation of FDCA with a polyol containing from 2 to 12 methylene groups: the longer the alkyl subunit, the higher the molecular mobility, the crystallization kinetics, and the ductility, and the lower the glass transition and melting temperatures [14,23,24,25,26]. The interest in this class of polyesters has recently raised not only for their fully bioderived nature, which allows for a considerable reduction in energy use and CO_2_ emissions in the production phase, but also for their outstanding mechanical, thermal, optical, and gas-barrier properties, which are comparable or even superior to those of the corresponding terephthalate counterparts [27]. 

Given their remarkable properties, PAFs are promising candidates to mitigate the drawbacks of PLA through physical blending, but despite the growing interest in this field, the corpus of studies on furanoate-based polymer blends in general, and PLA/PAF blends in particular, is still surprisingly limited. The most widely investigated blend is PLA/poly(butylene 2,5-furandicarboxylate) (PBF), reported as immiscible but with promising mechanical properties if the weight fraction of PBF is kept low (under 5 wt%) [28,29]. More recently, Terzopoulou et al. [30] synthesized a copolyester of PBF with poly(butylene adipate) (PBF-co-PBAd) and investigated its effectiveness as a compatibilizer for the PLA/PBF blends. A similar copolyester was instead used as the only additive by Wang et al. [31], who reported an increase in the strain at break from 5.7% of neat PLA to 222% of the blend containing 30 wt% of PBF-co-PBAd. 

Our group has recently carried out a detailed investigation on PLA/PAF blends prepared via solution mixing, for the production of bioderived films [32,33,34,35,36] and fibers [37,38,39]. That work involved blending PLA not only with PBF, but also with longer-alkyl-chain PAFs such as poly(pentamethylene furanoate) (PPeF), poly(hexamethylene furanoate) (PHF), poly(octamethylene furanoate) (POF), poly(decamethylene furanoate) (PDeF), and poly(dodecamethylene furanoate) (PDoF). These works confirmed that a small weight fraction of such PAFs enhance the strain at break and the fracture toughness of neat PLA. On the other hand, the scarce adhesion between the PAF domains and the surrounding PLA matrix suggested that even better properties could be obtained by adding a suitable compatibilizer. This was demonstrated in a subsequent work [40] on the development of PLA/PEF blends produced via melt mixing and compatibilized with a commercial compatibilizer/chain extender (Joncryl^®^ ADR 4468, J). In this work, this compatibilizer was proven to reduce the domain size of the dispersed phase and improve the interfacial adhesion. That work also evidenced the advantages of melt mixing over solution mixing. Although solution mixing allows for low-temperature processing and prevents undesired transesterification reactions typically observed with furanoate polyesters [23], it is poorly scalable and not environmentally friendly; moreover, residual solvents can remain in the samples and affect the resulting thermomechanical properties. In contrast, investigating the properties of PLA/PAF blends through melt mixing can produce industrially relevant results applicable in large-scale manufacturing plants.

Most of this recent research effort focuses on the development of PLA/PAF films for packaging applications, while very little has been done to produce PLA/PAF fibers, mostly via solution spinning. Conversely, only one work [41] has been found on the production of PLA/PEF fibers via melt spinning: in that work, the authors develop an interesting melt spinning variation that allows for the production of a nanofibrillar microstructure, but they lack the investigation of the mechanical properties of the resulting fibers. On the other hand, to the best of the authors’ knowledge, no works can be found in the open scientific literature on the thermomechanical properties of melt-spun PLA/PAF-compatibilized fiber blends including PAFs other than PEF. 

Therefore, this work aims, for the first time, at producing PLA/poly(propylene 2,5-furandicarboxylate) (PPF)- and PLA/PBF-compatibilized fibers via melt spinning and at understanding their morphological and thermomechanical properties as a function of the PAF type and weight fraction, also varying the collection speed. The PPF and PBF concentrations ranged between 0 and 20 wt%, while the concentration of the employed commercial compatibilizer (Joncryl^®^ ADR 4468, J) was fixed to 1 phr. The work first involved the melt blending of the constituents in an internal mixer to produce 2 mm thick sheets, which were characterized and then pelletized to produce single filaments through melt spinning. The characterization first involved a thorough rheological analysis of the blends, and then a detailed evaluation of the blends’ microstructure, thermal properties, and mechanical performance, both on bulk (sheets) and fiber samples. 

## 2. Results

### 2.1. Characterization of the Bulk (Sheet) Samples

#### 2.1.1. Rheological Properties

**Figure 1:**
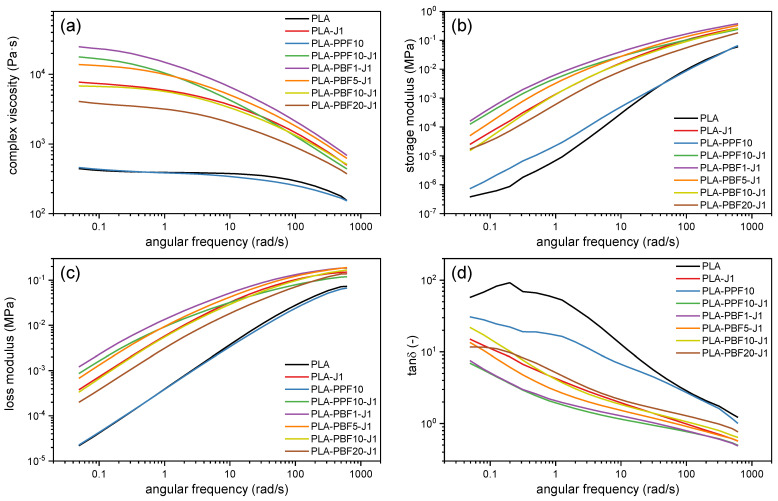
Results of dynamic rheological tests on some selected compositions. (**a**) Complex viscosity; (**b**) storage modulus; (**c**) loss modulus; (**d**) tanδ.

Dynamic rheological tests were carried out to assess the effect of the added PAF (PPF or PBF) and of the compatibilizer J on the rheological properties of PLA at the processing temperature (190 °C). Figure 1 shows the results of the dynamic rheological tests and reports the trends of the complex viscosity $\eta^{*}$, storage modulus ($G^{\prime }$), loss modulus ($G^{\prime \prime }$), and tanδ as a function of the applied frequency for some selected compositions. 

The complex viscosity of neat PLA is in line with what is reported in the literature for similar PLA grades [42,43], while the addition of 1 phr of J considerably increases the values of $\eta^{*}$ throughout the whole investigated frequency range. This implies that J is effective as a chain extender on neat PLA at the processing temperature investigated in this work. Moreover, the increased shear thinning sensitivity for PLA-J1, evidenced by the disappearance of the Newtonian plateau observed in neat PLA in favor of a more evident shear thinning behavior, is a clear sign of chain extension, enhanced entanglement, and possibly the formation of long-chain branching (LCB), as reported elsewhere for PLA/J systems [44]. This phenomenon was also appreciated during melt compounding by the observation of an increasing torque upon the addition of this chain extender.

The addition of 10 wt% of PPF does not modify the rheological behavior of PLA considerably. The slight increase in $G^{\prime }$ and decrease in $G^{\prime \prime }$ and tanδ, observed especially at low frequencies, may suggest some interfacial interaction between the two phases [45]. On the other hand, all samples containing both J and a PAF (either PPF or PBF) show a higher viscosity than neat PLA and, generally, rheological properties comparable with those of PLA-J1. More specifically, the blends containing a small (1–5 wt%) percentage of PAF exhibit a higher complex viscosity and $G^{\prime }$ than PLA-J1; this could be due to the contribution of the interfacial interaction of PLA and the PAF domains, which sums up to the branching and chain entanglement, as already observed in a previous work on PLA/PEF-compatibilized blends [40]. On the other hand, the increased percentage of PAF decreases the complex viscosity, due to a less effective compatibilization and, probably, the slightly lower viscosity of the neat PAFs. 

By comparing the samples PLA-PPF10-J1 and PLA-PBF10-J1, i.e., the two blends containing 10 wt% of either PPF or PBF, it can be observed that PPF seems to increase the viscosity, the storage modulus, and the loss modulus more than PBF, probably due to its higher own complex viscosity. 

Finally, it is worth noting that all the blends show a decreased tanδ compared to the neat PLA. Due to the chain extension and branching caused by J, but also to the interfacial interaction with the PAF domains, the rheological behavior of the melted blends manifests a higher melt elasticity. However, the values of tanδ are very rarely lower than 1, which highlights the persistence of a viscous, liquid-like behavior in most of the investigated frequency range. 

#### 2.1.2. Microstructural Properties of the Bulk (Sheet) Samples

**Figure 2:**
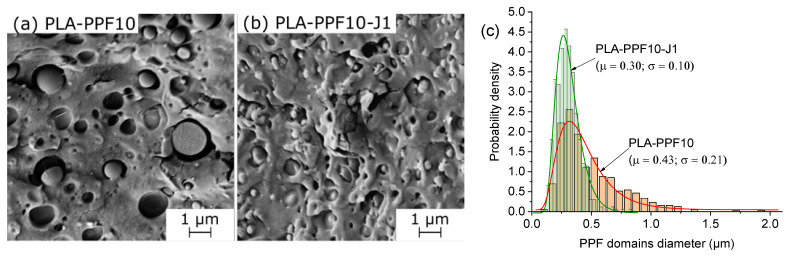
SEM micrographs of the cryofracture surface of the bulk samples (**a**) PLA-PPF10 and (**b**) PLA-PPF10-J1; (**c**) lognormal fitting of the PPF domain size of PLA-PPF10 and PLA-PPF10-J1.

A microstructural evaluation through SEM was performed to investigate the morphology of the prepared blends in terms of size, distribution, and interfacial compatibility. All samples show a sea–island morphology, which is a sign of blend immiscibility; however, as already observed for PLA/PEF blends [40], the addition of J considerably decreases the PAF domain size and improves the interfacial adhesion with the surrounding PLA matrix. This is evident by comparing the cryofracture surface of the samples PLA-PPF10 and PLA-PPF10-J1 (Figure 2a,b), which also shows the quantitative evaluation of the PPF domain size distribution (experimental data and log-normal fitting), clearly highlighting a decrease in the PPF domain size and a narrowing in the size distribution for the sample PLA-PPF10-J1. 

**Figure 3:**
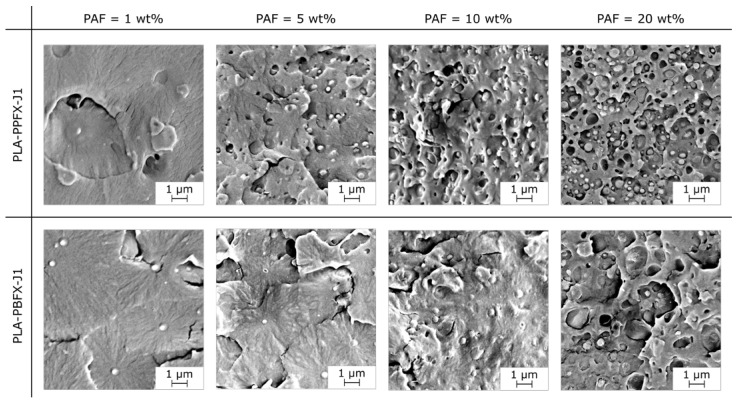
SEM micrographs of the cryofracture surface of the bulk samples PLA-PPFX-J1 and PLA-PBFX-J1 (x = 1–20 wt%).

The PAF domain size slightly increases with the PAF content, as shown in Figure 3 and reported in Table 1, which could be due to the coalescence of PAF droplets in the molten state. The PPF domain size increases from 0.25 µm for a PPF content of 1 wt% to 0.31 µm for a PPF concentration of 20 wt%, while the PBF domain size increases from 0.44 µm for a PBF amount of 1 wt% to 0.51 µm for a PBF content of 20 wt%. These data also show that the PBF domains are larger than those of PPF, probably due to a combination of different viscosity and compatibilization efficacy for these two polymers, but more studies are needed to fully clarify this concept.

**Figure 4:**
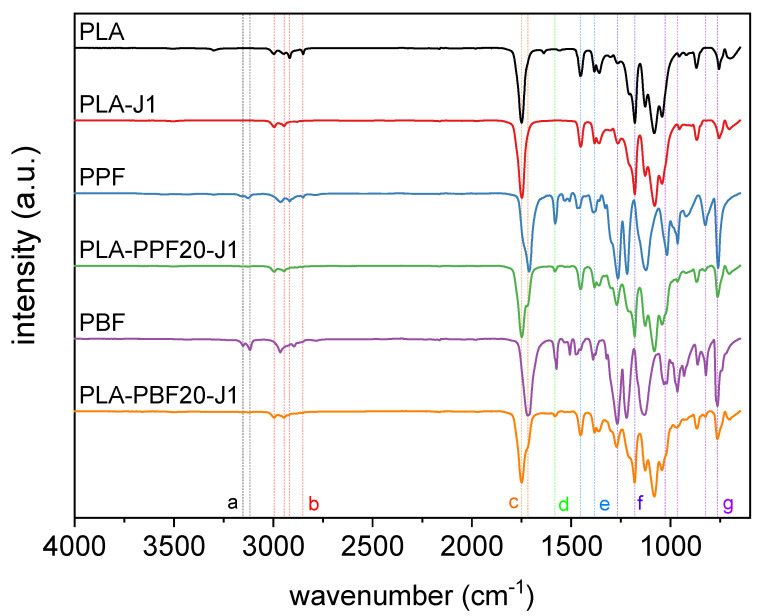
FT-IR spectra of some selected compositions. The letters refer to the peak assignment in Table 2.

The next step has been the evaluation of the prepared blends via FT-IR to highlight the possible occurrence of covalent bonding between PLA and the PAFs thanks to the addition of J. Figure 4 shows the FT-IR spectra of the neat PLA, PPF, and PBF and some selected blends, while the most important FT-IR signals are listed in Table 2 with the corresponding assignment. 

The spectrum of neat PLA shows a weak signal of the in-plane and out-of-plane C–H stretching vibration in the interval 2950–3000 cm^−1^, of the C=O stretching vibration at 1750 cm^−1^, and of the C–O–C stretching vibration at 1180 cm^−1^ [46]. On the other hand, neat PPF and PBF show the typical signals of furan-based aliphatic polyesters [47,48], i.e., the symmetric and asymmetric stretching of the furan ring at 3126/3117 and 3159/3152 cm^−1^; the symmetric and asymmetric C–H stretching at 2850–2960 cm^−1^; the vibration of the C=C bond of furan ring at 1574 cm^−1^ the ester carbonyl stretching at 1712/1718 cm^−1^ [49,50]; and the furan ring breathing and bending at 1018/1027 cm^−1^, 965 cm^−1^, 825 cm^−1^, and 760 cm^−1^. The spectra of the blends show the same vibrations of the two neat polymers, with only slight red or blue shifts in correspondence of only some signals, such as the C=O stretching, which might indicate some compatibilization. However, neither remarkable intensity variations nor the presence of new bands can be observed, which suggests that the interaction between PLA and the PAF domains is probably mostly due to weak intermolecular bonds rather than the formation of interphase covalent bonds. 

#### 2.1.3. Thermal Properties

**Figure 5:**
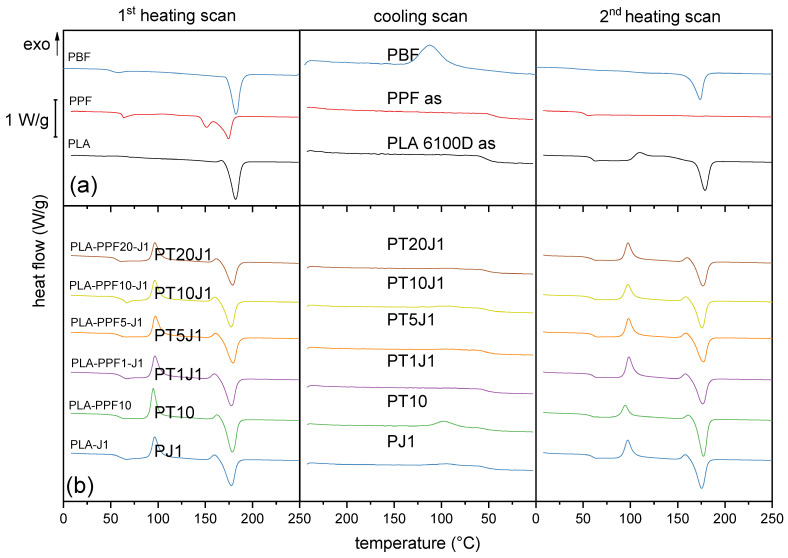
DSC thermograms (first heating scan, cooling scan, second heating scan) of some selected bulk compositions. (**a**) Granules of PLA, PPF, and PBF, as received; (**b**) bulk samples PLA-J1, PLA-PPF10, and PLA-PPFx-J1 (x = 1–20 wt%).

Figure 5a,b shows the DSC thermograms of neat PLA, PPF, and PBF (Figure 5a) as well as of PLA/PPF blends (Figure 5b), highlighting the thermal behavior of the samples in a heating–cooling–heating cycle (H1-C-H2). The thermograms of PLA/PBF blends, qualitatively similar to those shown here, are reported in the Supplementary Materials (Figure S1). 

The neat, as-received polymers present the traditional thermal profile of semicrystalline materials, with glass transition between 40 and 70 °C and melting/crystallization between 100 and 200 °C (Table 3). For all three polymers, the T_g_ in the second heating scan is approximately 10 °C lower than that measured in the first scan, because the first heating/cooling cycle erases the aging phenomena responsible for the T_g_ increase observed on the as-received materials. The T_g_ values measured on the three polyesters are in line with literature data [30,51,52], and in particular, it is interesting to note that, for the two furanoate polyesters, the slight lengthening of the alkyl subunit causes a remarkable decrease in T_g_ for PBF compared to PPF (40.7 °C vs. 51.6 °C in H2) [53]. In both cases, though, the T_g_ is lower than that of neat PLA (60.5 °C in H2). 

Moreover, all three polymers show an endothermic peak associated to melting in H1, with PPF and PBF exhibiting a similar T_m_ (173.3 °C vs. 178.4 °C) and crystallinity degree (39.5% vs. 47.8%). However, only PLA and PBF are capable of crystallizing in the cooling scan, and the crystallinity of PBF measured after the second heating scan is the highest, being 35.7%. Conversely, PPF is almost not capable of crystallizing while cooled at 10 °C/min; thus, it presents a very shallow melting event in H2 (∆H_m_ = 0.5 J/g). This is due to the following: (i) its relatively short alkyl subunit, which results in restricted chain mobility; and (ii) the odd number of methylene groups in this subunit, which further hinders the possibility of ordered chain arrangement. PBF, on the other hand, with four methylene groups, shows a higher tendency to crystallize. This is known as the odd-even effect, described for furanoate polyesters by Tsanaktsis et al. [54] and by Jiang et al. [55]. 

Since PLA/PPF and PLA/PBF blends were produced via melt mixing and compression molding, it is plausible that the microstructure and the crystallinity of the three polymer phases in the prepared blends resemble those found in the H2 scan performed on the parent polymers, and not the H1 stage. More specifically, PBF domains are likely to be semicrystalline, while the PPF phase is probably amorphous. This is supported by the DSC scans of the blends: the PLA/PPF blends (Figure 5b) do not show any melting event associated with PPF even in H1, and the measured melting temperature is constantly equal to that of PLA, while in PLA/PBF blends (Figure S1), the T_m_ measured in H1 decreases with an increase in PBF fraction, as observable also from the data reported in Table 4, because this melting event is the sum of the melting of the crystals of PLA and those of PBF. Due to the impossibility to distinguish the two contributions, the calculation of a crystallinity degree for PLA and PBF in the blends is not feasible. An attempt has been made to separate the two contributions by performing a DSC scan at a low heating rate (1 °C/min) on the blend PLA-PBF20-J1, but this has not been successful, as the peak, although narrower, is still single. 

Therefore, the value of X_c_ of the PLA phase can be calculated only for PLA/PPF blends, other than for neat PLA and PLA-J1, and is reported in Table 4. It can be observed that, for PLA, the X_c_ strongly decreases from 33.1% to 12.9% with the addition of J, due to its chain extension and branching effect. On the other hand, the introduction of PPF does not significantly modify the crystallinity degree of PLA, as all the PLA-PPFx-J1 blends show values of X_c_ in the range 10–14%.

Thermogravimetric analysis (TGA) was performed to assess the resistance of the prepared blends to thermal degradation. The TGA thermograms of the PLA/PBF blends are reported in the Supplementary Materials (Figure S2), while those of the PLA/PPF blends are not reported as they are qualitatively and quantitatively similar to those of the PLA/PBF samples. None of the samples show a mass loss at 100 °C, which indicates a very low moisture content and, hence, effective storage of the materials in dry conditions before testing. Neat PLA, PPF, and PBF degrade in a single step between 350 °C and 450 °C, with peak derivative thermogravimetry (DTG) temperatures (T_d_) of 383 °C, 400 °C, and 404 °C, respectively. Very similar values are found in the blends. These results are comparable to previous findings on PLA/PEF blends reported in a preceding study [40]. 

#### 2.1.4. Mechanical Properties

**Figure 6:**
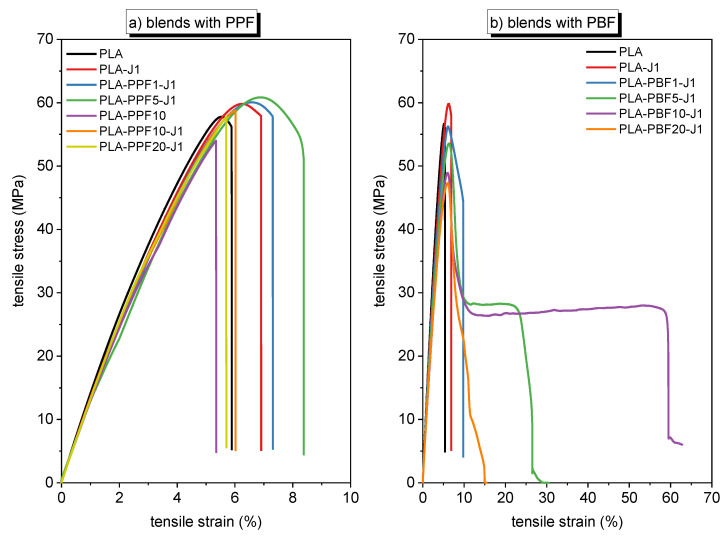
Representative stress–strain curves of the prepared compositions (bulk samples). (**a**) Blends with PPF; (**b**) blends with PBF.

Figure 6a,b shows representative tensile stress–strain curves on the samples PLA-PPFx-J1 and PLA-PBFx-J1 (x = 1–20 wt%), while the most important mechanical results are summarized in Table 5. Neat PLA shows an elastic modulus of approximately 4.0 GPa, a tensile strength of 56.3 MPa, and a strain at break of 5.2%, in line with semicrystalline PLAs of similar grade and in good agreement with the technical datasheet. As already observed in previous works [40], the addition of 1 phr J to neat PLA reduces the elastic modulus and increases the properties at break, in agreement with the decrease in the degree of crystallinity measured via DSC. 

For PLA/PPF blends (Figure 6a), the uncompatibilized blend PLA-PPF10 shows lower elastic modulus and similar ultimate tensile strength (UTS) and strain at break as the neat PLA, and the addition of J (sample PLA-PPF10-J1) increases the properties only modestly. The best ultimate properties among the PLA/PPF blends are shown by the sample PLA-PPF5-J1, which exhibits a stress at break of 59.8 MPa (+6% than neat PLA) and a strain at break of 7.7% (+48% than neat PLA), but these improvements are still modest. Although the stress–strain curves of the blends containing a small (<5 wt%) amount of PPF show incipient plasticization after the maximum stress, none of the specimens with this composition can sustain an extended necking propagation. 

Quite different is the case for PLA/PBF blends (Figure 6b)**,** where the samples with a PBF fraction of 5–10 wt% show a very evident yield point followed by a remarkable necking propagation accompanied by considerable whitening of the specimens, which results in a noteworthy increase in the strain at break. Samples PLA-PBF5-J1 and PLA-PBF10-J1, in fact, exhibit a similar elastic modulus and UTS as that of neat PLA, but their strain at break is 22% (+ 320% than neat PLA) and 55% (+ 960% than neat PLA), respectively. For PBF fractions lower than 5 wt% and higher than 10 wt%, the plasticization performance is more modest, even though a certain increase in the strain at break is still observable. These differences in the toughening ability of PPF and PBF on bulk PLA probably stem from the fact that PBF has a lower T_g_ and is tougher than PPF [29], which allows it to be more ductile and to effectively plasticize PLA, although the blend PLA/PPF seems better compatibilized, as the smaller domain size suggests (see SEM micrographs in Figure 3). 

### 2.2. Characterization of the Fiber Samples

#### 2.2.1. Microstructural Characterization

**Figure 7:**
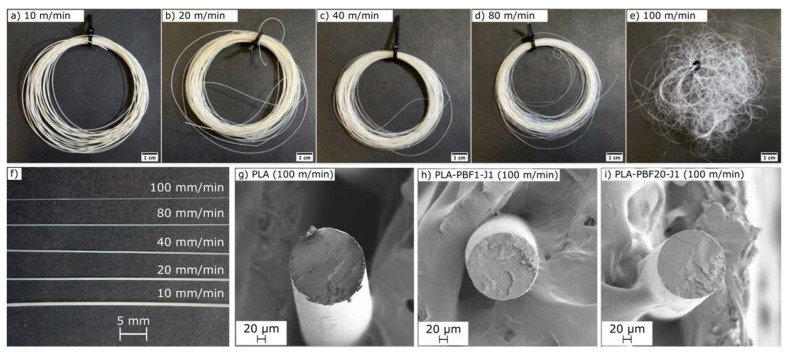
(**a**–**e**) Pictures of the fibers PLA-PBF5-J1 spun at different collection speeds; (**f**) comparison of the fibers PLA-PBF5-J1 to highlight the different diameters; (**g**–**i**) low magnification SEM micrograph of the PLA, PLA-PBF1-J1, and PLA-PBF20-J1 fibers collected at 100 m/min.

The compounded blends were pelletized and inserted in a single-screw extruder to produce fibers through melt spinning, with a take-up speed varying from 10 m/min to 100 m/min. As expected, the cross-section of the fibers decreases with the collection speed, as qualitatively observable from the pictures of the fibers PLA-PBF5-J1 shown in Figure 7a–f). All the produced fibers have a uniform and circular cross-section, regardless of the composition and the collection speed (see Figure 7g–i, reported as an example), and therefore it is possible to calculate the fiber diameters, reported in Table 6. The fiber diameter ranges from 87 µm to 570 µm and decreases with increasing collection speed, while it is not remarkably affected by the blend composition. From these diameters, the value of the spinning draw ratio (DR) was calculated as the ratio between the squared spinneret diameter (1 mm) and the squared fiber diameter [56]. The obtained DR values range from 3 to 132 (Table 6). Table 6 also reports the values of the fiber titers, which range from approximately 300 tex for the lowest take up speeds down to 5–20 tex for the highest take up speeds.

**Figure 8:**
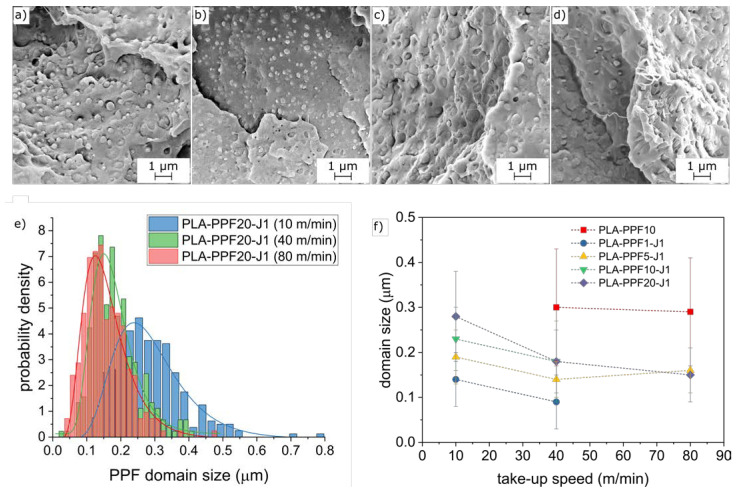
(**a**–**d**) SEM micrographs of the cryofracture surface of some selected fibers. (**a**) PLA-PPF20-J1 (10 m/min); (**b**) PLA-PPF20-J1 (80 m/min); (**c**) PLA-PBF20-J1 (10 m/min); (**d**) PLA-PBF20-J1 (80 m/min). (**e**) PPF domain size in fibers PLA-PPF20-J1 collected at different take-up speeds: experimental data and lognormal fitting; (**f**) PPF domain sizes in all PPF-containing fibers as a function of the take-up speed.

The increased take-up speed decreases not only the diameter of the fibers, but also the size of the PAF domains, as observable from Figure 8a–f, reporting the micrographs of the cryofracture surface of the fibers PLA-PPF20-J1 and PLA-PBF20-J1 collected at different take-up speeds (Figure 8a–d) and the domain size in the PLA-PPFx-J1 fibers (x = 1–20 wt%) (Figure 8e,f). All fibers present PAF domains smaller than those found in the bulk samples at the same PAF amount. Furthermore, the adhesion between the PAF domains and the surrounding PLA matrix seems improved compared to the bulk samples, probably because melt spinning, as an additional processing step in the melt, promotes a further reaction of J, thereby enhancing its compatibilizing effect. Moreover, the increase in the take-up speed further uniforms the microstructure by decreasing the domain size and narrowing the size distribution, as observable in Figure 8e,f for PPF-containing samples. All these effects contribute to the very interesting mechanical properties measured on the fibers, as described in Section 2.2.3.

#### 2.2.2. Thermal Characterization

All the prepared fibers were subjected to DSC and TGA tests with the same parameters as the bulk samples. For these tests, only the results of DSC on PLA/PBF fibers are reported in the Supplementary Materials (Figure S3a–f and Tables S1–S6) as an example. All the other data are not reported, as they are qualitatively similar to those of the bulk samples. They are only described qualitatively hereafter. TGA tests were performed especially to ensure that the fibers were properly stored in dry conditions, and the absence of any mass loss at 100 °C confirms this point. 

DSC scans were performed mainly to measure any impact of the spinning conditions on the crystallinity of the prepared blends. For the fibers PLA-PPFx-J1 (x = 1–20 wt%), the crystallinity of the PLA phase is generally higher than that measured on the bulk samples and slightly increases with the take-up speed, while it is not significantly affected by the PPF amount. The values of X_c_ range from 13–15% for the fibers collected at 10 m/min to 17–20% for the fibers collected at 80 m/min, probably due to the increased orientation of the polymer chains. For the samples PLA-PBFx-J1 (x = 1–20 wt%), the quantification of the crystallinity is more difficult due to the very close melting events of PLA and PBF (see Section 2.1.3). However, the melting enthalpy ΔH_m_ slightly increases with the collection speed, passing from 45–46 J/g of the fibers collected at 10 m/min to 48–50 J/g of the fibers collected at 80–100 m/min. This suggests that, also for these samples, the crystallinity slightly increases with the collection speed. Nevertheless, since the extra drawing to which the fibers are subjected is applied to the supercooled melt just after the extrusion, the impact on crystallinity and chain orientation is, expectedly, rather modest, and the two main effects of the increased collection speed are the decrease in diameter and the microstructural homogenization. 

#### 2.2.3. Mechanical Characterization

**Figure 9:**
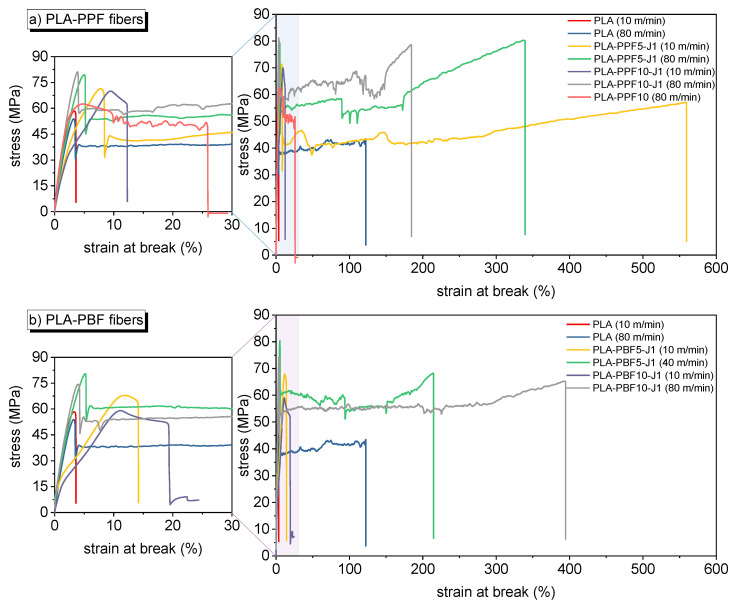
Representative stress–strain curves of some selected fiber compositions. (**a**) PLA-PPF fibers; (**b**) PLA-PBF fibers.

**Figure 10:**
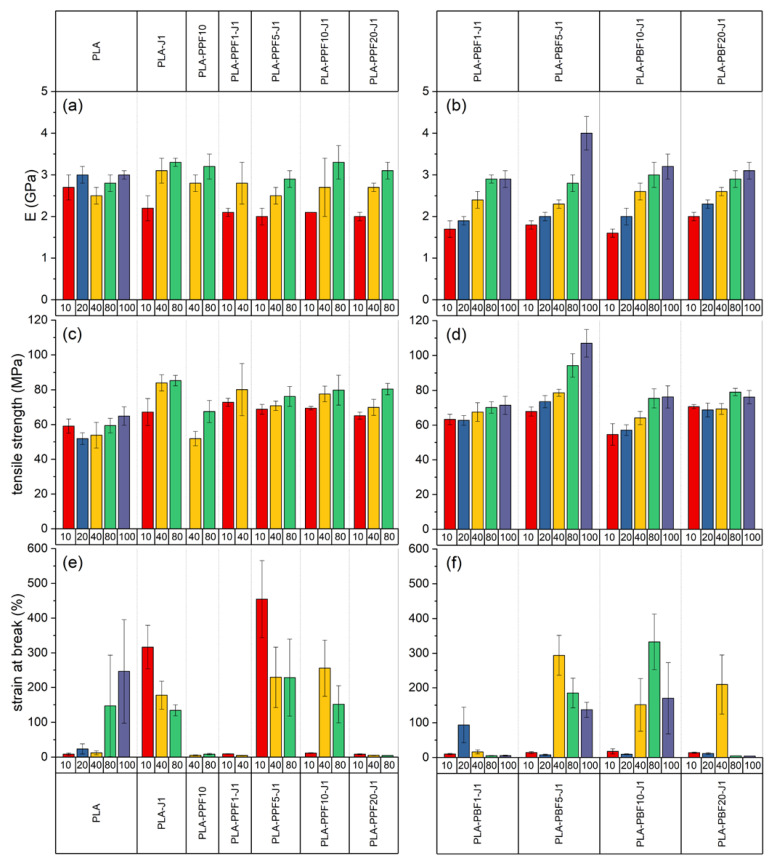
Mechanical properties of the prepared fiber samples. Numbers on the x axes represent the collection speed in m/min. (**a**,**b**) Elastic modulus; (**c**,**d**) tensile strength; (**e**,**f**) strain at break.

Figure 9 shows representative tensile stress–strain curves of some selected fiber compositions, while the most important mechanical parameters extracted from these tests are reported in Figure 10. Neat PLA fibers exhibit an elastic modulus of 2.5–3 GPa and a mechanical strength of approximately 60 MPa, regardless of the collection speed, while the strain at break is 3–10% for low take-up speeds and increases up to 246% for fibers collected at 100 m/min, probably due to the scarce chain orientation coupled with the size effect. The addition of J further improves the strain at break, likely due to the chain extension effect performed by J on PLA. 

What is more interesting, though, is the effect of PPF and PBF on the mechanical properties. An increased PPF and PBF fraction, in fact, increases the elastic modulus and the mechanical strength, and this effect is more evident for PBF-containing fibers collected at higher take-up speeds. Moreover, some compositions also present an exceptional strain at break; for example, the strain at break is equal to 333% for the sample PLA-PBF10-J1 collected at 80 m/min and to 293% for the sample PLA-PBF5-J1 collected at 40 m/min. These results confirm the plasticizing effect performed by a small amount of PBF on PLA observed on bulk samples. What is even more remarkable is that the same plasticizing effect is now observed also for PPF; for instance, the composition PLA-PPF5-J1 collected at 10 m/min shows a noticeable strain at break of 455%, which is 50 times higher than that of neat PLA collected at the same collection speed, but also other samples containing 5–10 wt% of PPF show strain at break higher than 200%. Additionally, the presence of a considerable strain-hardening region after the yield point for some compositions suggests the possibility of a subsequent cold drawing, to further increase the mechanical properties of these fibers. 

**Figure 11:**
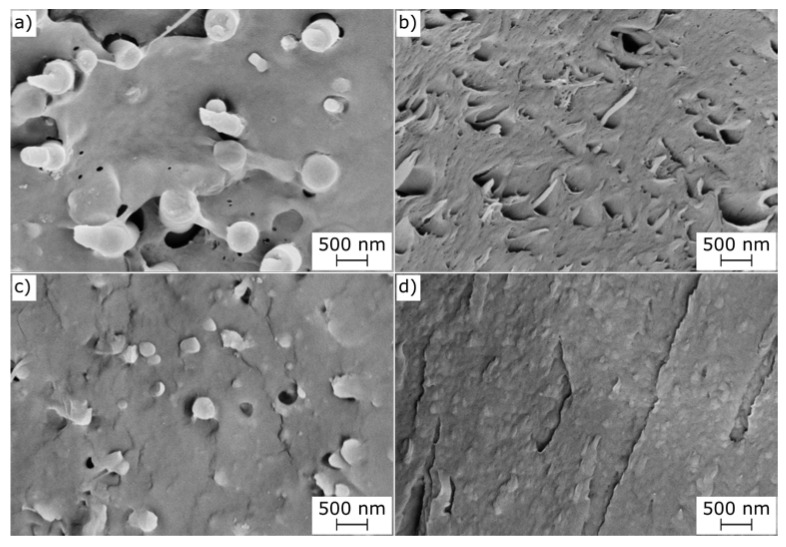
Comparison between the cryofracture surface (**a**–**c**) and the tensile ductile fracture surface (**b**–**d**) of the following: (**a**,**b**) PLA-PPF10 (80 m/min); (**c**,**d**) PLA-PPF10-J1 (40 m/min).

This toughening effect is probably due to the higher microstructural homogenization resulting from the fiber spinning, the smaller PPF and PBF domain size, and the enhancement of the effect of J, which probably completes its reaction in the melt spinning stage. All these effects favor the load transfer between the PLA matrix and the PAF domains, which can sustain the load and effectively toughen the resulting fibers. In fact, the SEM micrographs of the tensile fracture surface of the PPF fibers (Figure 11) clearly show that the PPF domains considerably deform in the tensile direction, and their shape changes from spheres to fibrils.

It is worth mentioning that the non-compatibilized PLA-PPF10 sample collected at 80 m/min showed a ductile behavior for 4 samples out of 10, with a strain at break of 35.1%. Additionally, these samples showed considerable deformation of the PPF domains, as shown in Figure 11b, but the adhesion between these domains and the surrounding PLA matrix was poorer than that observed on the compatibilized samples (Figure 11d). 

This deformation may be caused by a possible “plastic–rubber” transition during the tensile test, where the heat caused by the internal friction during the test may induce the local glass-to-rubber transition of PPF and PBF and cause their softening. This effect has already been observed for PLA/PBF bulk samples [29], but this is the first time that it has been observed on PLA/PBF fibers and especially on PLA/PPF samples. Given the higher T_g_ of PPF compared to PBF, the observation of this phenomenon on PPF is even more remarkable. This considerable orientation of the PPF and PBF domains may also induce their crystallization, thereby causing a strengthening–toughening combined effect on PLA. This may explain the concurrent increase in the tensile strength and elastic modulus observed for some compositions. 

## 3. Materials and Methods

### 3.1. Materials

Fiber-grade PLA Ingeo^®^ 6100D was provided by NatureWorks LLC (Minnetonka, MN, USA) in form of granules. It shows a density of 1.24 g/cm^3^ and a melt index of 24 g/10 min at 210 °C. High-molecular-weight poly(propylene 2,5-furandicarboxylate) (PPF) and poly(butylene 2,5-furandicarboxylate) (PBF) were synthesized via the two-stage melt polycondensation method (esterification and polycondensation) in a glass batch reactor as described in our previous works [57,58]. In brief, for the preparation of the polyesters, proper amounts of 2,5-dimethylfuran-dicarboxylate (DMFD) and the appropriate diols at a molar ratio of diester/diol = 1/2 were charged into the reaction tube of the polyesterification apparatus. TBT (400 ppm) was added as catalyst, and the apparatus with the reagents was evacuated several times and filled with argon in order to remove the whole oxygen amount. The reaction mixture was heated at 160 °C under argon atmosphere (5 mL/min) for 2 h, at 170 °C for additional 2 h, and finally at 180–200 °C for 2 h. This first step (transesterification) was considered completed after the collection of almost all the theoretical amount of CH_3_OH, which was removed from the reaction mixture by distillation and collected in a graduate cylinder. In the second step of polycondensation, a vacuum (5.0 Pa) was applied slowly over a period of time of about 15 min to remove the excess of diols and to avoid excessive foaming. as well as to minimize oligomer sublimation, which is a potential problem during the melt polycondensation. The temperature was increased during vacuum application to 220 °C, while the stirring speed was increased to 720 rpm. The reaction continued at this temperature for 1 h, and after that time, the temperature was increased to 235 °C for 2 h and to 250 °C for an additional 2 h. After the polycondensation reactions were completed, the polyesters were easily removed, milled, and washed with methanol. Intrinsic viscosity (*η*) measurements of prepared polyesters were performed using an Ubbelohde viscometer at 30 °C in a mixture of phenol/1,1,2,2-tetrachloroethane (60/40 *w*/*w*), and it was found that their (*η*) values are as follows: PBF = 0.68 dL/g (Mn = 18,166 g/mol) and PPF = 0.63 dL/g (Mn = 16,150 g/mol). 

The employed compatibilizer/chain extender was a poly(styrene-acrylic-co-glycidyl methacrylate) (SAGMA), commercially known as Joncryl^®^ ADR 4468 (J). It was supplied by BASF Gmbh (Ludwigshafen am Rhein, Germany) in the form of colorless solid flakes and used as received. It shows a density of 1.08 g/cm^3^ and a glass transition temperature of 59 °C, according to the producer’s datasheet. 

### 3.2. Sample Preparation

#### 3.2.1. Bulk Samples

Samples based on PLA/PPF- and PLA/PBF-compatibilized blends were produced via melt compounding and hot pressing. PLA, PPF, PBF, and J granules were dried overnight at 80 °C in vacuum conditions and added in a proper amount into a melt compounder Thermo Haake Rheomix 600, where they were processed at 190 °C for 10 min, with a rotational speed of the counter-rotating rotors of 50 rpm (J was added at minute 3, after complete melting all polymer phases). The melt-compounded material was then hot-pressed with a Carver hot plate press at 190 °C for 10 min, with a load of 7 tons applied on a square mold of 120 × 120 × 2 mm^3^ (applied pressure = 4.8 MPa). 

The nominal compositions of the prepared blends, in terms of the relative amount of PPF, PBF, and J, are reported in Table 7. The amount of PPF and PBF ranged from 1 to 20 wt%, as previous studies on PLA/PAF blends showed that the optimal PAF amount that optimized the mechanical properties fell in this range [40]. For the compatibilized blends, the amount of J was fixed to 1 phr, as this was selected as the optimal composition based on previous experiments on similar PLA/PAF systems [40]. However, one uncompatibilized blend containing 10 wt% of PPF (i.e., PLA-PPF10) was produced to verify the effectiveness of J as a compatibilizer. The uncompatibilized blend of PLA-PBF10 was not produced due to the scarcity of material available. However, the advantage of adding a proper compatibilizer was amply demonstrated by verifying the scarce mechanical properties of the samples PLA-PPF10 (this work) and PLA-PEF10 (containing 10 wt% of poly(ethylene furanoate), characterized in a previous work of our group [40]). 

#### 3.2.2. Fiber Samples

Fiber samples were prepared through melt compounding and melt spinning. Melt compounding was performed with the same parameters adopted for the preparation of bulk samples. After compounding, the blends were cryogenically grinded with the aid of liquid nitrogen in an IKA M20 universal blade mill (IKA, Staufen, Germany), for a total of 10 min in steps of 2 min each, to avoid excessive heat generation. Prior to the melt spinning process, the obtained coarse powders were dried overnight at 80 °C under vacuum conditions. 

**Figure 12:**
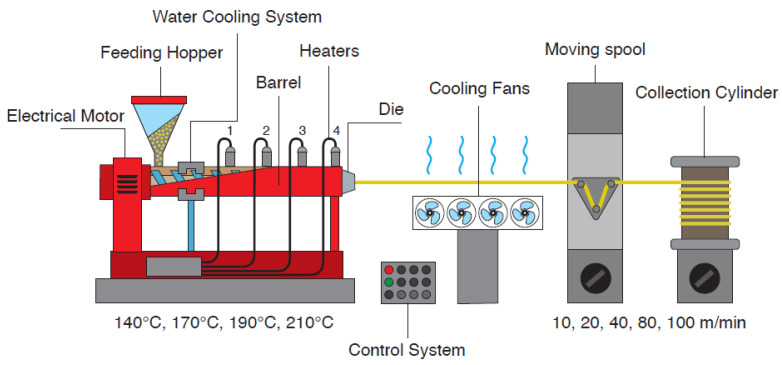
Schematic representation of the melt spinning apparatus, constituted by different components (single-screw extruder, cooling fans, moving spool, and collection cylinder).

Melt spinning was performed in a Friuli Filiere (Udine, Italy) TCM500 single-screw laboratory extruder, equipped with a screw of 14 mm diameter (L/D ratio = 20) and a single nozzle circular spinneret with a diameter of 1 mm. A representation of the laboratory extruder along with the main components of the melt spinning apparatus and the most important spinning parameters (temperature profile and collection rates) are presented in Figure 12. The instrument had temperature control through four different heating bands along the extrusion cylinder (one of which on the spinneret), and a cooling jacket in the feeding zone to avoid immediate melting of the material and possible clogging of the cylinder. Two sets of cooling fans were placed in series with the extruder, one immediately after the die and another set of four at 30 cm from the die. The collection system was made of a moving spool that directed the fibers and a rotating drum for the collection; two different cylinders were employed (respectively with a diameter equal to 5 cm and 20 cm), depending on the required collection rates.

The rotational speed of the screw was set to 25 rpm, and the four different temperatures along the barrel were set at 140, 170, 190, and 210 °C. This temperature profile was chosen, after some preliminary trials, as a trade-off between the optimal profile for the blends containing 1 wt% and 20 wt% of furanoate polyester, i.e., the blends with the highest and lowest viscosity, respectively, as highlighted by the rheological tests performed before spinning. The choice of the processing temperatures was further supported by other studies from the literature about the melt spinning of both PLA Ingeo^®^ Biopolymer 6100D [59] and its blends [60], with a single-screw extruder set at an ascending temperature profile ranging approximately between 180 °C and 220 °C. 

The three selected collection rates applied on all compositions were 10 m/min, 40 m/min, and 80 m/min. Some samples could also be collected at 100 m/min, but only for the PBF-containing blends. Some preliminary trials were also performed by collecting the fibers at 20 m/min. The samples were labelled as the bulk samples with the additional indication of the collection speed. For instance, the sample PLA_PBF20_J1_40 m/min is the composition containing 20 wt% of PBF, 1 phr of Joncryl, and collected at 40 m/min. 

### 3.3. Characterization

The rheological properties of the prepared blends were investigated with a Discovery HR-2 hybrid rheometer (TA instrument, New Castle, DE, USA), in a parallel-plate configuration (gap = 1.9 mm), in frequency sweep mode from 0.05 to 600 rad/s at 190 °C. The tests were performed on disc specimens that were laser-cut from the prepared sheets (diameter = 25 mm, thickness = 2 mm). This test allowed for the measurement of the complex viscosity $\eta^{*},$ the shear storage and loss moduli ($G^{\prime },G^{\prime \prime }$), and the tanδ as a function of frequency. 

The microstructural properties of the prepared blends were analyzed via a field-emission scanning electron microscope (FE-SEM) Zeiss Supra 40 (Carl Zeiss AG, Oberkochen, Germany) by analyzing the cryofracture surface after Pt-Pd sputtering. 

Fourier-transform infrared (FT-IR) spectroscopy was performed in attenuated total reflectance (ATR) mode on the surface of the prepared samples via a Perkin-Elmer Spectrum One IR spectrometer (Perkin Elmer GmbH, Waltham, MA, USA), equipped with a ZnSe crystal. In total, 100 scans were superimposed for each spectrum in a wavenumber range of 650–4000 cm^−1^ (resolution = 4 cm^−1^). 

Thermogravimetric analysis (TGA) was performed with a Mettler TG50 thermobalance (Mettler Toledo, Columbus, OH, USA). Specimens of approximately 20 mg were cut from the prepared sheets and subjected to a thermal ramp of 10 °C/min from room temperature up to 700 °C, under a nitrogen flow of 20 mL/min. One specimen was tested per composition. The tests allowed for measuring the temperatures associated to a mass loss of 1 wt%, 3 wt%, and 5 wt% ($T_{1\%,}T_{3\%,}T_{5\%}$), the onset degradation temperature ($T_{onset}$) via the tangent method, the degradation temperature ($T_{d}$), corresponding to the peak of the derivative thermogravimetry (DTG) curve, and the final mass after the test ($m_{r}$).

Differential scanning calorimetry (DSC) was carried out with a Mettler DSC30 calorimeter (Mettler Toledo, Columbus, OH, USA) on specimens of approximately 15 mg cut from the prepared sheets. The specimens were subjected to a heating–cooling–heating cycle at 10 °C/min between 0 °C and 250 °C, under a nitrogen flow of 100 mL/min. One specimen was measured per each composition. The test resulted in the determination of the glass transition temperature ($T_{g}$) and the melting, crystallization, and cold crystallization temperatures and enthalpies ($T_{m,}\;T_{c,}\;T_{cc,}\;H_{m,}\;H_{c,}\;H_{cc}$) of the PLA phase in the blends. Where possible, the degree of crystallinity of PLA, PPF, and PBF ($\chi_{PLA},\;\chi_{PPF},\;\chi_{PBF}$) was calculated via Equation (1) [61]:

(1) $$\chi =\frac{{\Delta H}_{m}-{\Delta H}_{cc}}{{\Delta H}_{0}\cdot \omega }\cdot 100$$

where ${∆H}_{0}$ is the theoretical melting enthalpy, equal to 93.7 J/g for PLA [62] and 129 J/g for PBF [63], respectively, and *ω* is the weight fraction of PLA or PEF.

The mechanical properties of the prepared blends were investigated, for bulk sheet samples, on dumbbell 1BA specimens (UNI EN ISO 527-2) via quasi-static tensile tests, performed on an Instron 5969 universal testing dynamometer (Norwood, MA, USA), equipped with a 1 kN load cell. The elastic modulus (E) was determined by testing five specimens at 0.25 mm/min while measuring the strain with a resistance extensometer Instron 2620, having a gauge length of 12.5 mm. The elastic modulus was evaluated as the slope of the stress–strain curve between the strain levels of 0.05% and 0.25%. Five additional specimens were tested at 1 mm/min until rupture, and these properties allowed for the measurement of the ultimate tensile strength (UTS), evaluated as the maximum stress and the strain at break ($\varepsilon_{b}$).

Mechanical tensile tests were also performed on all the collected fiber samples. In this case, the Instron 5969 was equipped with a 100 N load cell. To perform mechanical tensile tests on fibers, 10 specimens per sample were prepared gluing the fibers onto paper frames with a gauge length of 20 mm, to facilitate their handling. The as-spun fibers were mounted in the tensile testing grips, fixed at a distance of 20 mm, and tested at 10 mm/min till break. The elastic modulus, ultimate tensile strength, and strain at break were evaluated as described for the bulk samples. All the mechanical tests were performed at a temperature of 25 °C and with a relative humidity of 40%. 

## 4. Conclusions

This work demonstrated the successful preparation of PLA/PPF and PLA/PBF blends in the form of bulk (sheet) samples and, for the first time, also in the form of melt-spun fibers. The detailed physical, thermal, and mechanical characterization highlighted the positive contribution of PPF and PBF in tuning PLA performance as a function of the PAF concentration (1 to 20 wt%) and compatibilization. 

For bulk samples, although all the prepared blends showed a sea–island morphology, indicating blend immiscibility, the addition of 1 phr of a commercial compatibilizer reduced the size of PAF domains and improved the interfacial adhesion with the PLA matrix. Conversely, the PAF domain size slightly increased with increasing PAF content, possibly due to coalescence of PAF droplets during melting, and PBF domains were larger than those of PPF, likely due to differences in viscosity and compatibilization efficacy between the two furanoate polyesters. The immiscibility between PLA and either of the considered PAFs was also evident from the DSC analysis, in which the T_g_ measured on the blends was similar to that of PLA. Moreover, the slow crystallization rate of PPF results in amorphous PPF domains, while the PBF domains are likely semicrystalline. 

For the mechanical properties, PLA-PPF5-J1 demonstrated the highest stress at break of 59.8 MPa (+6% compared to neat PLA) and a strain at break of 7.7% (+48% compared to PLA). PLA/PBF blends with 5–10 wt% PBF exhibited a distinct yield point, followed by remarkable necking propagation, resulting in a significant increase in strain at break. Samples PLA-PBF5-J1 and PLA-PBF10-J1 displayed similar elastic modulus and UTS to neat PLA, but with strain at break values of 22% (+320% compared to neat PLA) and 55% (+960% compared to neat PLA), respectively. Hence, although the PLA/PPF blends appeared better when compatibilized, as evident from smaller domain size in SEM micrographs, PBF exhibited an improved toughening abilities on bulk PLA, likely due to PBF’s lower T_g_ and greater toughness.

Quite different are the results of the mechanical tests on the prepared fibers. Increasing the PPF and PBF fraction resulted in higher elastic modulus and mechanical strength, particularly for PBF-containing fibers collected at higher take-up speeds. Notably, certain compositions showed exceptional strain at break, such as PLA-PBF10-J1 collected at 80 m/min (strain at break = 333%) and PLA-PBF5-J1 collected at 40 m/min (strain at break = 293%), which confirmed the plasticizing effect of PBF observed in bulk samples. Interestingly, a similar plasticizing effect was observed for PPF, with the PLA-PPF5-J1 composition collected at 10 m/min exhibiting a remarkable strain at break of 455%, which was significantly higher than neat PLA collected at the same speed. 

These differences can be explained by the fact that the fiber spinning process promoted microstructural homogenization, resulting in smaller PPF and PBF domains. Additionally, the impact of J was enhanced, potentially completing its reaction during melt spinning. These effects improved load transfer between the PLA matrix and PAF domains, effectively toughening PLA. SEM analysis revealed considerable deformation of PPF domains in the tensile direction, indicating their contribution to the toughness of the material. The presence of a “plastic–rubber” transition during the tensile test, induced by internal friction and heat, may explain the softening and deformation of PPF and PBF. The orientation of PPF and PBF domains could also induce their own crystallization, contributing to the simultaneous increase in tensile strength and elastic modulus observed in some compositions.

Overall, this work demonstrated the potential of PPF and PBF in tuning the thermo-mechanical properties of PLA, both in the bulk and fiber form, and expanded the potential applications of these biopolymers in the packaging and textile fields. 

## Figures and Tables

**Table 1:** Diameter of the PAF domains in the bulk samples PLA-PPFX-J1 and PLA-PBFX-J1 (x = 1–20 wt%).

PAF wt%	PPF Domain Size (µm)	PBF Domain Size (µm)
1	0.25 + 0.10	0.44 + 0.11
5	0.31 + 0.12	0.43 + 0.14
10	0.30 + 0.10	0.50 + 0.10
20	0.31 + 0.14	0.51 + 0.15

**Table 2:** Assignment of the main FT-IR peaks of some selected samples.

Assignment	Label	PLA	PPF	PLA-PPF20-J1	PBF	PLA-PBF20-J1
$\nu_{as}$ CH (Fu)	A	/	3159	/	3152	/
$\nu_{s}$ CH (Fu)		/	3126	/	3117	/
$\nu_{as}$ CH_3_	B	2996	/	2995	/	2995
$\nu_{s}$ CH_3_		2945	/	2945	/	2945
$\nu_{as}$ CH		2919	2963	2921	2964	2921
$\nu_{s}$ CH		2852	2850	/	2894	2851
ν C=O	C	1750	1712	1750/1712	1718	1749/1721
ν C=C (Fu)	D	/	1581	/	1574	1582
$\delta_{as}$ CH_3_	E	1453	1453	1453	1453	1458
$\delta_{as}$ CH_3_		1383	1389	1383	1389	1383
ν C–O	F	1180	1265	1181	1268/1121	1273/1180
Fu ring breathing	G	/	1018	/	1027	/
Fu ring bending		/	965; 825; 760	965; 825; 760	965; 822; 763	966; 823; 764

**Table 3:** Main results of DSC tests on as received granules of PLA, PPF, and PBF.

	PLA			PPF			PBF		
Thermal Cycle	H1	C	H2	H1	C	H2	H1	C	H2
T_g_ [°C]	71.8	50.7	60.5	62.0	45.2	51.6	50.0	/	40.7
T_cc_ [°C]	/	/	111.0	105.1	/	/	/	/	/
∆H_cc_ [J/g]	/	/	36.9	3.3	/	/	/	/	/
T_m_ [°C]	186.7	/	179.4	173.3	/	170.6	178.4	/	170.8
∆H_m_ [J/g]	51.8	/	42.8	59.3	/	0.5	61.6	/	46.1
T_c_ [°C]	/	/	/	/	/	/	/	114.7	/
∆H_c_ [J/g]	/	/	/	/	/	/	/	61.6	/
X_c_ [%]	55.2	/	6.3	39.5	/	0.4	47.8	/	35.7

H1 = first heating scan, C = cooling scan, H2 = second heating scan, T_g_ = glass transition temperature, T_cc_ = cold crystallization temperature, ∆H_cc_ = cold crystallization enthalpy, T_m_ = melting temperature, ∆H_m_ = melting enthalpy, T_c_ = crystallization temperature, ∆H_c_ = crystallization enthalpy, and X_c_ = crystallinity degree.

**Table 4:** Main results of DSC test (first heating scan—H1 and cooling scan—C) on the prepared blends (bulk samples).

Sample	T_g_ (°C)	T_cc_ (°C)	∆H_cc_ (J/g)	T_m_ (°C)	∆H_m_ (J/g)	X_c_ (PLA) (%)	Tc (°C)	∆Hc (J/g)
PLA	59.9	94.7	26.3	183.2	57.3	33.1	95.8	10.9
PLA-J1	56.7	96.7	31.2	176.1	43.2	12.9	94.8	3.8
PLA-PPF10	58.3	95.3	37.3	176.8	51	16.2	98.7	12.9
PLA-PPF1-J1	61.3	97.2	32.3	175.6	45.3	14.2	/	/
PLA-PPF5-J1	59.3	97.4	32.1	178.2	42	11.2	/	/
PLA-PPF10-J1	54.7	96.8	28.2	176.8	37.3	10.9	/	/
PLA-PPF20-J1	54.7	96.8	28.2	176.8	37.3	12.3	/	/
PLA-PBF1-J1	58.9	97	31.5	178.9	43.5	/	96.1	3.8
PLA-PBF5-J1	62.8	96	31.9	177.9	45	/	97.8	17
PLA-PBF10-J1	39.3/59.0	97.1	29.7	177.9	45.2	/	95	5
PLA-PBF20-J1	39.5/59.2	96.7	28.3	178.5	45.3	/	99.4	9.8

T_g_ = glass transition temperature, T_cc_ = cold crystallization temperature, ∆H_cc_ = cold crystallization enthalpy, T_m_ = melting temperature, ∆H_m_ = melting enthalpy, T_c_ = crystallization temperature, ∆H_c_ = crystallization enthalpy, and X_c_ = crystallinity degree.

**Table 5:** Main properties obtained from the tensile tests on the prepared bulk samples.

Sample	E (GPa)	UTS (MPa)	ε_b_ (%)
PLA	4.0 ± 0.1	56.3 ± 0.4	5.2 ± 0.1
PLA-J1	3.1 ± 0.1	60.7 ± 0.8	6.6 ± 0.4
PLA-PPF1-J1	2.7 ± 0.1	60.2 ± 0.9	7.2 ± 0.4
PLA-PPF5-J1	2.5 ± 0.1	59.8 ± 1.3	7.7 ± 0.8
PLA-PPF10	3.0 ± 0.1	54.7 ± 1.0	5.3 ± 0.3
PLA-PPF10-J1	3.1 ± 0.1	58.5 ± 2.8	6.2 ± 0.9
PLA-PPF20-J1	2.6 ± 0.1	56.1 ± 4.3	5.6 ± 0.7
PLA-PBF1-J1	3.4 ± 0.1	55.5 ± 0.5	8.3 ± 1.1
PLA-PBF5-J1	3.3 ± 0.1	53.4 ± 1.9	22.0 ± 1.5
PLA-PBF10-J1	3.4 ± 0.2	49.7 ± 1.0	55.5 ± 24.5
PLA-PBF20-J1	2.9 ± 0.1	44.8 ± 3.7	11.5 ± 3.1

E = elastic modulus; UTS = ultimate tensile strength; ε_b_ = strain at break.

**Table 6:** Diameters (expressed in μm) and titers (expressed in tex) of the fiber samples prepared at different collection speeds (average ± standard deviation). Values in parentheses represent the draw ratios.

Diameters (µm)	10 m/min	20 m/min	40 m/min	80 m/min	100 m/min
PLA	569 ± 22 (3.1)	347 ± 21 (8.3)	302 ± 14 (11.0)	207 ± 38 (23.3)	151 ± 21 (43.9)
PLA-J1	457 ± 70 (4.8)	/	219 ± 15 (20.9)	184 ± 13 (29.5)	/
PLA-PPF10	/	/	225 ± 15 (19.8)	134 ± 12 (55.7)	/
PLA-PPF1-J1	485 ± 25 (4.3)	/	235 ± 24 (18.1)	/	/
PLA-PPF5-J1	449 ± 34 (5.0)	/	232 ± 19 (18.6)	209 ± 30 (22.9)	/
PLA-PPF10-J1	497 ± 22 (4.0)	/	242 ± 18 (17.1)	179 ± 19 (31.2)	/
PLA-PPF20-J1	500 ± 20 (4.0)	/	250 ± 11 (16.0)	178 ± 9 (31.6)	/
PLA-PBF1-J1	497 ± 54 (4.0)	339 ± 34 (8.7)	241 ± 17 (17.2)	163 ± 14 (37.6)	151 ± 11 (43.9)
PLA-PBF5-J1	521 ± 38 (3.7)	326 ± 21 (9.4)	265 ± 12 (14.2)	186 ± 18 (28.9)	87 ± 23 (132.1)
PLA-PBF10-J1	566 ± 32 (3.1)	414 ± 18 (5.8)	303 ± 32 (10.9)	205 ± 32 (23.8)	120 ± 32 (69.4)
PLA-PBF20-J1	557 ± 28 (3.2)	389 ± 10 (6.6)	281 ± 11 (12.7)	202 ± 7 (24.5)	157 ± 8 (40.6)
**Titer (tex)**	**10 m/min**	**20 m/min**	**40 m/min**	**80 m/min**	**100 m/min**
PLA	321 ± 3	154 ± 9	88 ± 4	39 ± 8	30 ± 2
PLA-J1	240 ± 49	/	99 ± 13	75 ± 12	/
PLA-PPF10	/	/	49 ± 5	20 ± 2	/
PLA-PPF1-J1	219 ± 7	/	57 ± 7	/	/
PLA-PPF5-J1	196 ± 9	/	47 ± 5	41 ± 8	/
PLA-PPF10-J1	241 ± 6	/	58 ± 4	46 ± 2	/
PLA-PPF20-J1	263 ± 12	/	62 ± 6	30 ± 1	/
PLA-PBF1-J1	259 ± 10	140 ± 4	62 ± 8	31 ± 2	24 ± 2
PLA-PBF5-J1	256 ± 9	110 ± 10	69 ± 3	35 ± 5	5 ± 1
PLA-PBF10-J1	319 ± 5	177 ± 7	84 ± 10	44 ± 3	23 ± 7
PLA-PBF20-J1	311 ± 11	155 ± 6	83 ± 6	42 ± 2	25 ± 3

**Table 7:** List of the prepared samples with nominal weight compositions.

Label	PLA (wt%)	PPF (wt%)	PBF (wt%)	J (phr)
PLA	100	0	0	0
PLA-J1	100	0	0	1
PLA_PPF10	90	10	0	0
PLA_PPF1_J1	99	1	0	1
PLA_PPF5_J1	95	5	0	1
PLA_PPF10_J1	90	10	0	1
PLA_PPF20_J1	80	20	0	1
PLA_PBF1_J1	99	0	1	1
PLA_PBF5_J1	95	0	5	1
PLA_PBF10_J1	90	0	10	1
PLA_PBF20_J1	80	0	20	1

## Data Availability

Data are available on request by the corresponding author.

## Supplementary Materials

The following supporting information can be downloaded at: https://www.mdpi.com/article/10.3390/molecules28124811/s1.

## Sample Availability

Samples of the produced compounds are available from the authors.

## References

[1] Meraldo A., Wagner J.R. (2016). 4—Introduction to Bio-Based Polymers. Multilayer Flexible Packaging.

[2] Geyer R., Jambeck J.R., Law K.L. (2017). Production, use, and fate of all plastics ever made. Sci. Adv..

[3] White E., Bassilakis R., Nogués S. (2020). From the Plastics Present to a Sustainable Future: The Bioplastics Innovation Landscape, Players and Market Opportunities.

[4] Fredi G., Dorigato A. (2021). Recycling of bioplastic waste: A review. Adv. Ind. Eng. Polym. Res..

[5] Konstantopoulou M., Terzopoulou Z., Nerantzaki M., Tsagkalias J., Achilias D.S., Bikiaris D.N., Exarhopoulos S., Papageorgiou D.G., Papageorgiou G.Z. (2017). Poly(ethylene furanoate-co-ethylene terephthalate) biobased copolymers: Synthesis, thermal properties and cocrystallization behavior. Eur. Polym. J..

[6] Niaounakis M. (2013). Biopolymers Reuse, Recycling, and Disposal.

[7] Yang Y., Zhang M., Ju Z., Tam P.Y., Hua T., Younas M.W., Kamrul H., Hu H. (2020). Poly(lactic acid) fibers, yarns and fabrics: Manufacturing, properties and applications. Text. Res. J..

[8] Liu Z., Wang Y., Wu B., Cui C., Guo Y., Yan C. (2019). A critical review of fused deposition modeling 3D printing technology in manufacturing polylactic acid parts. Int. J. Adv. Manuf. Technol..

[9] Pegoretti A., Fambri L., Migliaresi C. (1997). In Vitro degradation of poly(L-lactic acid) fibers produced by melt spinning. J. Appl. Polym. Sci..

[10] Tait M., Pegoretti A., Dorigato A., Kaladzidou K. (2011). The effect of filler type and content and the manufacturing process on the performance of multifunctional carbon/poly-lactide composites. Carbon.

[11] Balla E., Daniilidis V., Karlioti G., Kalamas T., Stefanidou M., Bikiaris N.D., Vlachopoulos A., Koumentakou I., Bikiaris D.N. (2021). Poly(lactic Acid): A Versatile Biobased Polymer for the Future with Multifunctional Properties—From Monomer Synthesis, Polymerization Techniques and Molecular Weight Increase to PLA Applications. Polymers.

[12] Fambri L., Pegoretti A., Fenner R., Incardona S.D., Migliaresi C. (1997). Biodegradable fibres of poly(L-lactic acid) produced by melt spinning. Polymer.

[13] Fambri L., Bragagna S., Migliaresi C. (2006). Biodegradable Fibers of Poly-L, DL-lactide 70/30 Produced by Melt Spinning. Macromol. Symp..

[14] Sousa A.F., Patrício R., Terzopoulou Z., Bikiaris D.N., Stern T., Wenger J., Loos K., Lotti N., Siracusa V., Szymczyk A. (2021). Recommendations for replacing PET on packaging, fiber, and film materials with biobased counterparts. Green Chem..

[15] Zhang Z., Zhou J., Yu S., Wei L., Hu Z., Xiang H., Zhu M. (2023). Melt-spun bio-based PLA-co-PET copolyester fibers with tunable properties: Synergistic effects of chemical structure and drawing process. Int. J. Biol. Macromol..

[16] Perego G., Cella G.D., Bastioli C. (1996). Effect of molecular weight and crystallinity on poly(lactic acid) mechanical properties. J. Appl. Polym. Sci..

[17] Labrecque L.V., Kumar R.A., Dave V., Gross R.A., McCarthy S.P. (1997). Citrate esters as plasticizers for poly(lactic acid). J. Appl. Polym. Sci..

[18] Portes dos Santos T., Dias K.B., Bischoff E., Santos Mauler R. (2022). Synthesis of esters derived from 2,5-furandicarboxylic acid and study of its plasticizing effects on poly(lactic acid). J. Polym. Res..

[19] Cabedo L., Feijoo J.L., Villanueva M.P., Lagaron J.M., Gimenez E. (2006). Optimization of Biodegradable Nanocomposites Based on aPLA/PCL Blends for Food Packaging Applications. Macromol. Symp..

[20] Wang L., Ma W., Gross R.A., McCarthy S.P. (1998). Reactive compatibilization of biodegradable blends of poly(lactic acid) and poly(ε-caprolactone). Polym. Degrad. Stab..

[21] Xu H., Zhou J., Odelius K., Guo Z., Guan X., Hakkarainen M. (2021). Nanostructured Phase Morphology of a Biobased Copolymer for Tough and UV-Resistant Polylactide. ACS Appl. Polym. Mater..

[22] Bozell J.J., Petersen G.R. (2010). Technology development for the production of biobased products from biorefinery carbohydrates—The US Department of Energy’s “Top 10” revisited. Green Chem..

[23] Poulopoulou N., Smyrnioti D., Nikolaidis G.N., Tsitsimaka I., Christodoulou E., Bikiaris D.N., Charitopoulou M.A., Achilias D.S., Kapnisti M., Papageorgiou G.Z. (2020). Sustainable Plastics from Biomass: Blends of Polyesters Based on 2,5-Furandicarboxylic Acid. Polymers.

[24] Sousa A.F., Silvestre A.J.D. (2022). Plastics from renewable sources as green and sustainable alternatives. Curr. Opin. Green Sustain. Chem..

[25] Eerhart A.J.J.E., Faaij A.P.C., Patel M.K. (2012). Replacing fossil based PET with biobased PEF; Process analysis, energy and GHG balance. Energy Environ. Sci..

[26] Paszkiewicz S., Walkowiak K., Irska I., Mechowska S., Stankiewicz K., Zubkiewicz A., Piesowicz E., Miadlicki P. (2022). Influence of the Multiple Injection Moulding and Composting Time on the Properties of Selected Packaging and Furan-Based Polyesters. J. Polym. Environ..

[27] Nguyen H.T.H., Qi P., Rostagno M., Feteha A., Miller S.A. (2018). The quest for high glass transition temperature bioplastics. J. Mater. Chem. A.

[28] Poulopoulou N., Kantoutsis G., Bikiaris D.N., Achilias D.S., Kapnisti M., Papageorgiou G.Z. (2019). Biobased Engineering Thermoplastics: Poly(butylene 2,5-furandicarboxylate) Blends. Polymers.

[29] Long Y., Zhang R.Y., Huang J.C., Wang J.G., Jiang Y.H., Hu G.H., Yang J., Zhu J. (2017). Tensile Property Balanced and Gas Barrier Improved Poly(lactic acid) by Blending with Biobased Poly(butylene 2,5-furan dicarboxylate). ACS Sustain. Chem. Eng..

[30] Terzopoulou Z., Zamboulis A., Papadopoulos L., Grigora M.E., Tsongas K., Tzetzis D., Bikiaris D.N., Papageorgiou G.Z. (2022). Blending PLA with Polyesters Based on 2,5-Furan Dicarboxylic Acid: Evaluation of Physicochemical and Nanomechanical Properties. Polymers.

[31] Wang G., Zhang L., Wang J., Hao X., Dong Y., Sun R. (2022). Ductile polylactic acid-based blend derived from bio-based poly(butylene adipate-co-butylene furandicarboxylate). Polym. Bull..

[32] Fredi G., Dorigato A., Bortolotti M., Pegoretti A., Bikiaris D.N. (2020). Mechanical and Functional Properties of Novel Biobased Poly(decylene-2,5-furanoate)/Carbon Nanotubes Nanocomposite Films. Polymers.

[33] Rigotti D., Soccio M., Dorigato A., Gazzano M., Siracusa V., Fredi G., Lotti N. (2021). Novel biobased polylactic acid/poly(pentamethylene 2,5-furanoate) blends for sustainable food packaging. ACS Sustain. Chem. Eng..

[34] Fredi G., Jafari M.K., Dorigato A., Bikiaris D.N., Pegoretti A. (2022). Improving the Thermomechanical Properties of Poly(lactic acid) via Reduced Graphene Oxide and Bioderived Poly(decamethylene 2,5-furandicarboxylate). Materials.

[35] Fredi G., Jafari M.K., Dorigato A., Bikiaris D.N., Checchetto R., Favaro M., Brusa R.S., Pegoretti A. (2021). Multifunctionality of reduced graphene oxide in bioderived polylactide/poly(dodecylene furanoate) nanocomposite films. Molecules.

[36] Fredi G., Rigotti D., Bikiaris D.N., Dorigato A. (2021). Tuning thermo-mechanical properties of poly(lactic acid) films through blending with bioderived poly(alkylene furanoate)s with different alkyl chain length for sustainable packaging. Polymer.

[37] Perin D., Rigotti D., Fredi G., Papageorgiou G.Z., Bikiaris D.N., Dorigato A. (2021). Innovative bio-based poly(lactic acid)/poly(alkylene furanoate) fiber blends for sustainable textile applications. J. Polym. Environ..

[38] Perin D., Fredi G., Rigotti D., Lotti N., Dorigato A. (2022). Sustainable textile fibers made of bioderived polylactide/poly(pentamethylene 2,5-furanoate) blends. J. Appl. Polym. Sci..

[39] Rigotti D., Fredi G., Perin D., Bikiaris D.N., Pegoretti A., Dorigato A. (2022). Statistical Modeling and Optimization of the Drawing Process of Bioderived Polylactide/Poly(Dodecylene Furanoate) Wet-Spun Fibers. Polymers.

[40] Fredi G., Dorigato A., Dussin A., Xanthopoulou E., Bikiaris D.N., Botta L., Fiore V., Pegoretti A. (2022). Compatibilization of polylactide/poly(ethylene furanoate) (PLA/PEF) blends for sustainable and bioderived packaging. Molecules.

[41] Zhu X., Ren Q., Li W., Wu M., Weng Z., Wang J., Zheng W., Wang L. (2022). In situ nanofibrillar fully-biobased poly (lactic acid)/poly (ethylene 2,5-furandicarboxylate) composites with promoted crystallization kinetics, mechanical properties, and heat resistance. Polym. Degrad. Stab..

[42] Mysiukiewicz O., Barczewski M., Skorczewska K., Matykiewicz D. (2020). Correlation between Processing Parameters and Degradation of Different Polylactide Grades during Twin-Screw Extrusion. Polymers.

[43] Peinado V., Castell P., Garcia L., Fernandez A. (2015). Effect of Extrusion on the Mechanical and Rheological Properties of a Reinforced Poly(Lactic Acid): Reprocessing and Recycling of Biobased Materials. Materials.

[44] Yahyaee N., Javadi A., Garmabi H., Khaki A. (2019). Effect of Two-Step Chain Extension using Joncryl and PMDA on the Rheological Properties of Poly (lactic acid). Macromol. Mater. Eng..

[45] Kahraman Y., Alkan Goksu Y., Özdemir B., Eker Gümüş B., Nofar M. (2021). Composition design of PLA/TPU emulsion blends compatibilized with multifunctional epoxy-based chain extender to tackle high impact resistant ductile structures. J. Appl. Polym. Sci..

[46] Mofokeng J.P., Luyt A.S., Tábi T., Kovács J. (2011). Comparison of injection moulded, natural fibre-reinforced composites with PP and PLA as matrices. J. Thermoplast. Compos. Mater..

[47] Xie H., Wu L., Li B.-G., Dubois P. (2018). Poly(ethylene 2,5-furandicarboxylate-mb-poly(tetramethylene glycol)) multiblock copolymers: From high tough thermoplastics to elastomers. Polymer.

[48] Sanusi O.M., Papadopoulos L., Klonos P.A., Terzopoulou Z., Hocine N.A., Benelfellah A., Papageorgiou G.Z., Kyritsis A., Bikiaris D.N. (2020). Calorimetric and Dielectric Study of Renewable Poly(hexylene 2,5-furan-dicarboxylate)-Based Nanocomposites In Situ Filled with Small Amounts of Graphene Platelets and Silica Nanoparticles. Polymers.

[49] Matos M., Sousa A.F., Andrade M., Silva N.H.C.S., Freire C.S.R., Mendes A., Silvestre A.J.D. (2018). Furanoate-Based Nanocomposites: A Case Study Using Poly(Butylene 2,5-Furanoate) and Poly(Butylene 2,5-Furanoate)-co-(Butylene Diglycolate) and Bacterial Cellulose. Polymers.

[50] Gomes M., Gandini A., Silvestre A.J.D., Reis B. (2011). Synthesis and characterization of poly(2,5-furan dicarboxylate)s based on a variety of diols. J. Polym. Sci. Part A Polym. Chem..

[51] Hu H., Zhang R., Shi L., Ying W.B., Wang J., Zhu J. (2018). Modification of Poly(butylene 2,5-furandicarboxylate) with Lactic Acid for Biodegradable Copolyesters with Good Mechanical and Barrier Properties. Ind. Eng. Chem. Res..

[52] Paszkiewicz S., Janowska I., Pawlikowska D., Szymczyk A., Irska I., Lisiecki S., Stanik R., Gude M., Piesowicz E. (2018). New functional nanocomposites based on poly(trimethylene 2,5-furanoate) and few layer graphene prepared by in situ polymerization. Express Polym. Lett..

[53] Papageorgiou D.G., Guigo N., Tsanaktsis V., Exarhopoulos S., Bikiaris D.N., Sbirrazzuoli N., Papageorgiou G.Z. (2016). Fast Crystallization and Melting Behavior of a Long-Spaced Aliphatic Furandicarboxylate Biobased Polyester, Poly(dodecylene 2,5-furanoate). Ind. Eng. Chem. Res..

[54] Tsanaktsis V., Terzopoulou Z., Nerantzaki M., Papageorgiou G.Z., Bikiaris D.N. (2016). New poly(pentylene furanoate) and poly(heptylene furanoate) sustainable polyesters from diols with odd methylene groups. Mater. Lett..

[55] Jiang Y., Woortman A.J.J., Alberda van Ekenstein G.O.R., Loos K. (2015). A biocatalytic approach towards sustainable furanic–aliphatic polyesters. Polym. Chem..

[56] Fambri L., Dabrowska I., Ceccato R., Pegoretti A. (2017). Effects of Fumed Silica and Draw Ratio on Nanocomposite Polypropylene Fibers. Polymers.

[57] Papageorgiou G.Z., Tsanaktsis V., Papageorgiou D.G., Exarhopoulos S., Papageorgiou M., Bikiaris D.N. (2014). Evaluation of polyesters from renewable resources as alternatives to the current fossil-based polymers. Phase transitions of poly(butylene 2,5-furan-dicarboxylate). Polymer.

[58] Papageorgiou G.Z., Papageorgiou D.G., Tsanaktsis V., Bikiaris D.N. (2015). Synthesis of the bio-based polyester poly(propylene 2,5-furan dicarboxylate). Comparison of thermal behavior and solid state structure with its terephthalate and naphthalate homologues. Polymer.

[59] Bruckmoser K., Resch K. (2015). Effect of processing conditions on crystallization behavior and mechanical properties of poly(lactic acid) staple fibers. J. Appl. Polym. Sci..

[60] Pivsa-Art W., Pivsa-Art S. (2020). Multifilament yarns of polyoxymethylene/poly(lactic acid) blends produced by a melt-spinning method. Text. Res. J..

[61] Sperling L.H. (2006). Introduction to Physical Polymer Science.

[62] Garlotta D. (2001). A Literature Review of Poly(Lactic Acid). J. Polym. Environ..

[63] Papageorgiou G.Z., Papageorgiou D.G., Terzopoulou Z., Bikiaris D.N. (2016). Production of bio-based 2,5-furan dicarboxylate polyesters: Recent progress and critical aspects in their synthesis and thermal properties. Eur. Polym. J..

