# Supplementary material for: Toughening Effect of 2,5-Furandicaboxylate Polyesters on Polylactide-Based Renewable Fibers
Source: Molecules. 2023 Jun 16;28(12):4811. doi: 10.3390/molecules28124811 (PMC10304103; doi:10.3390/molecules28124811)
Supplement: Supplementary file 1 [file molecules-28-04811-s001.zip › molecules-2443633-supplementary.pdf]

Supplementary materials

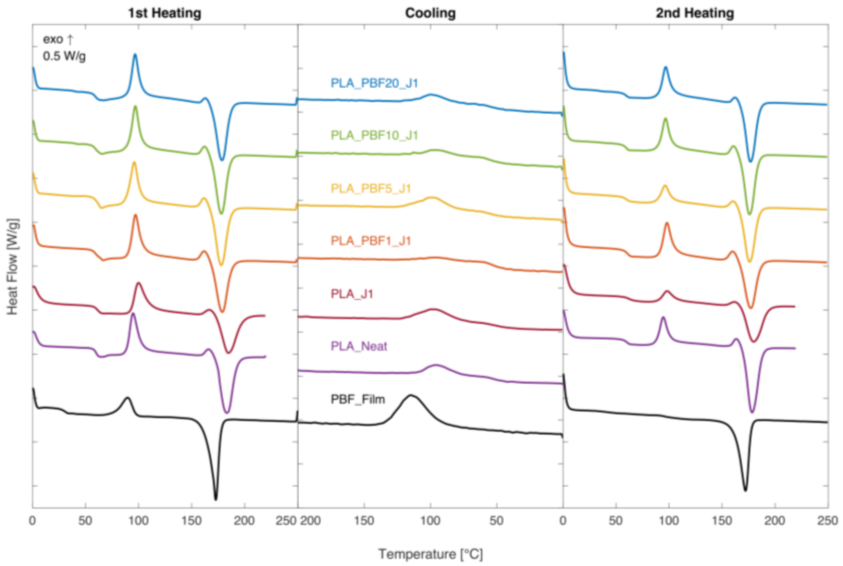

Figure S1. DSC thermograms (first heating scan, cooling scan, second heating scan) of some selected bulk compositions. PLA, PBF (film), PLA-J1, and PLA-PBFx-J1 (x = 1 – 20).

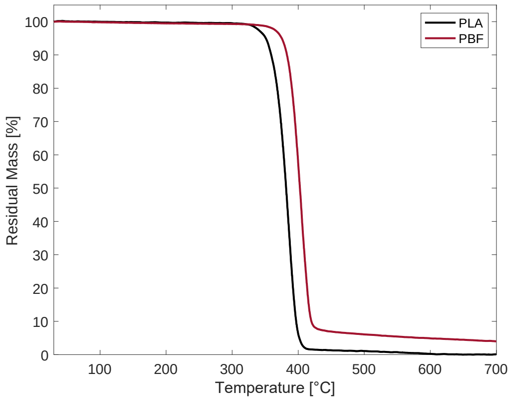

(**a**)

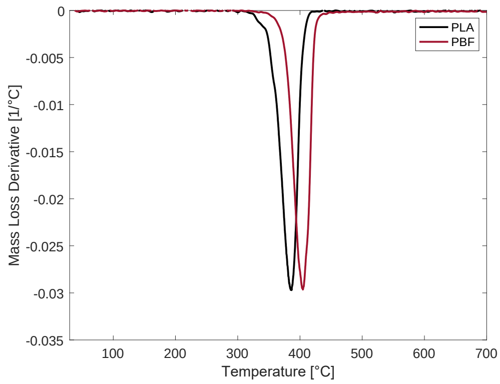

(**b**)

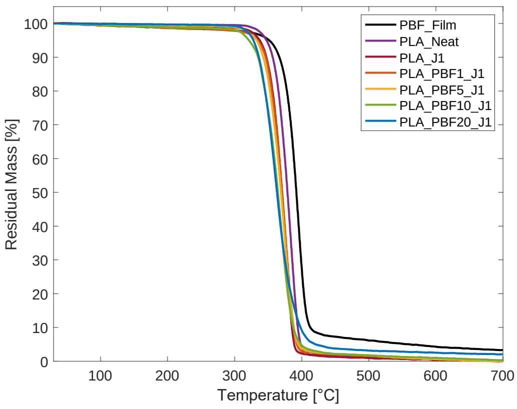

(**c**)

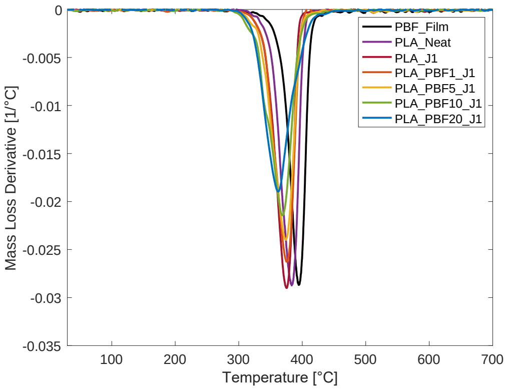

(**d**)

Figure S2. (**a**,**b**) TGA thermograms of as received PLA and PBF: (**a**) residual mass and (**b**) mass loss derivative (DTG) as a function of temperature. (**c**,**d**) TGA thermograms of PLA-PBFx-J1 bulk samples and PBF film: (**c**) residual mass and (**d**) mass loss derivative (DTG) as a function of temperature.

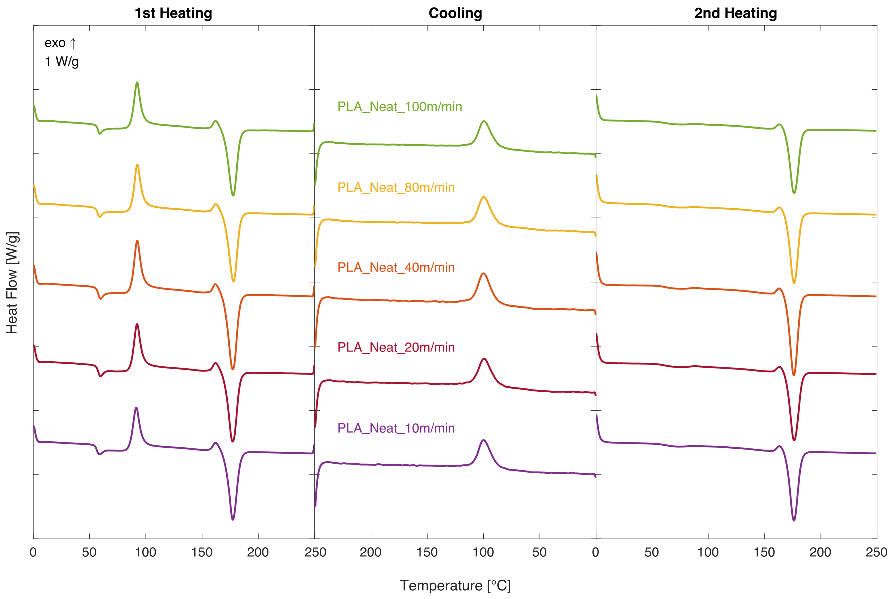
1st Heating
Cooling
2nd Heating
exo ↑
1 W/g
PLA_Neat_100m/min
PLA_Neat_80m/min
PLA_Neat_40m/min
PLA_Neat_20m/min
PLA_Neat_10m/min
Heat Flow [W/g]
0 50 100 150 200 250 200 150 100 50 0 50 100 150 200 250
Temperature [°C]

(**a**)

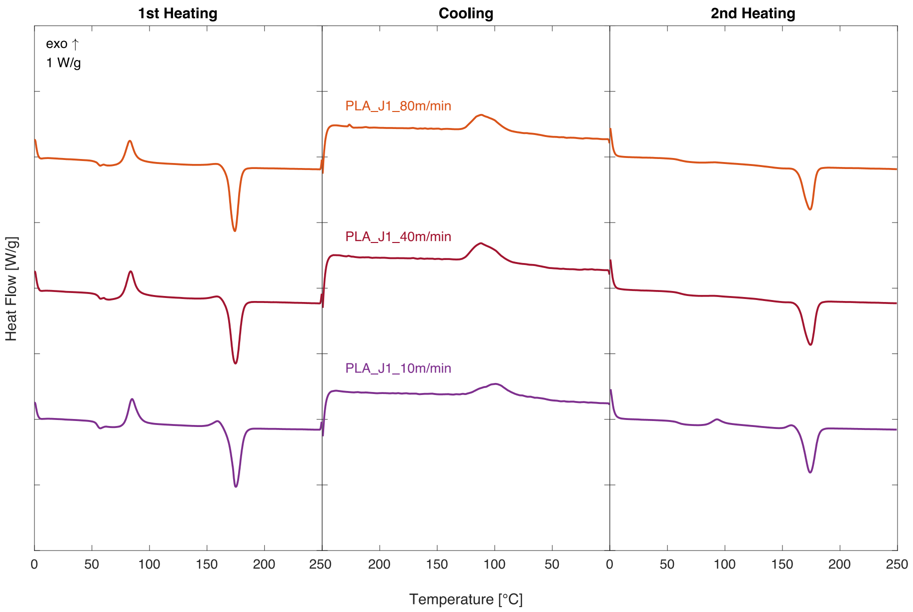
1st Heating
Cooling
2nd Heating
exo ↑
1 W/g
PLA_J1_80m/min
PLA_J1_40m/min
PLA_J1_10m/min
Heat Flow [W/g]
0 50 100 150 200 250 200 150 100 50 0 50 100 150 200 250
Temperature [°C]

(**b**)

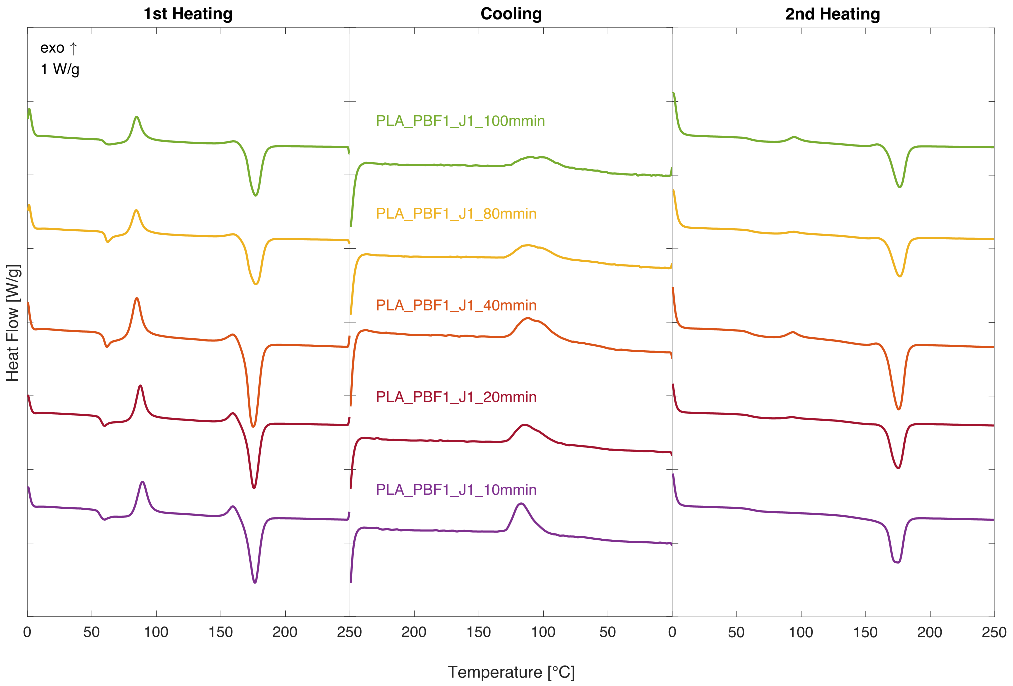
1st Heating
Cooling
2nd Heating
exo ↑
1 W/g
Heat Flow [W/g]
PLA_PBF1_J1_100mmin
PLA_PBF1_J1_80mmin
PLA_PBF1_J1_40mmin
PLA_PBF1_J1_20mmin
PLA_PBF1_J1_10mmin
0
50
100
150
200
250
200
150
100
50
0
50
100
150
200
250
Temperature [°C]

(**c**)

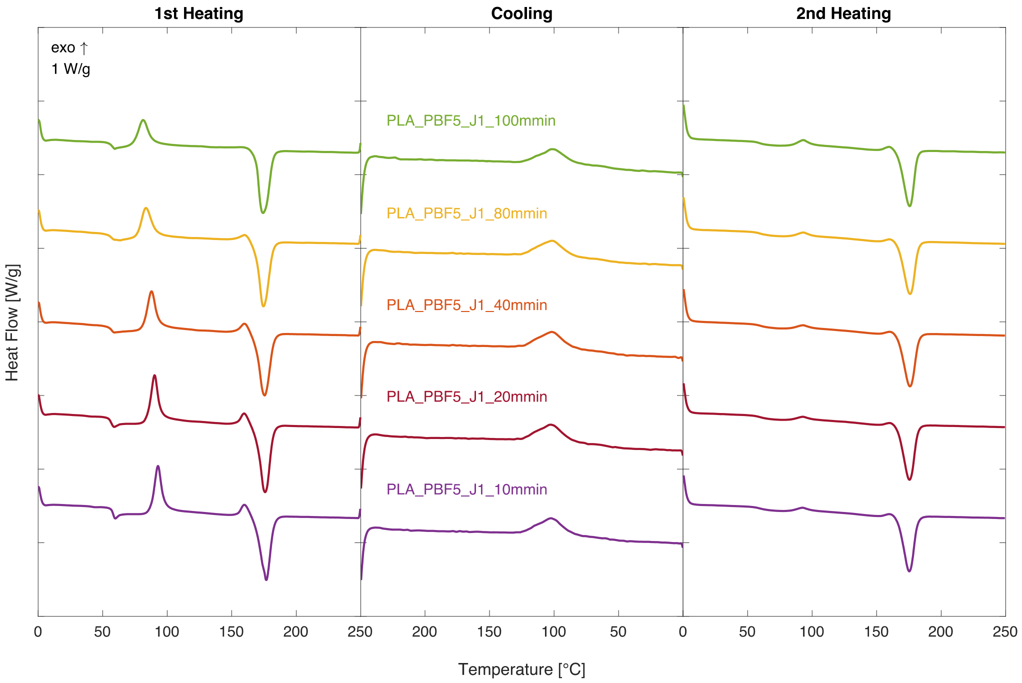
1st Heating
Cooling
2nd Heating
exo ↑
1 W/g
Heat Flow [W/g]
PLA_PBF5_J1_100mmin
PLA_PBF5_J1_80mmin
PLA_PBF5_J1_40mmin
PLA_PBF5_J1_20mmin
PLA_PBF5_J1_10mmin
0
50
100
150
200
250
200
150
100
50
0
50
100
150
200
250
Temperature [°C]

(**d**)

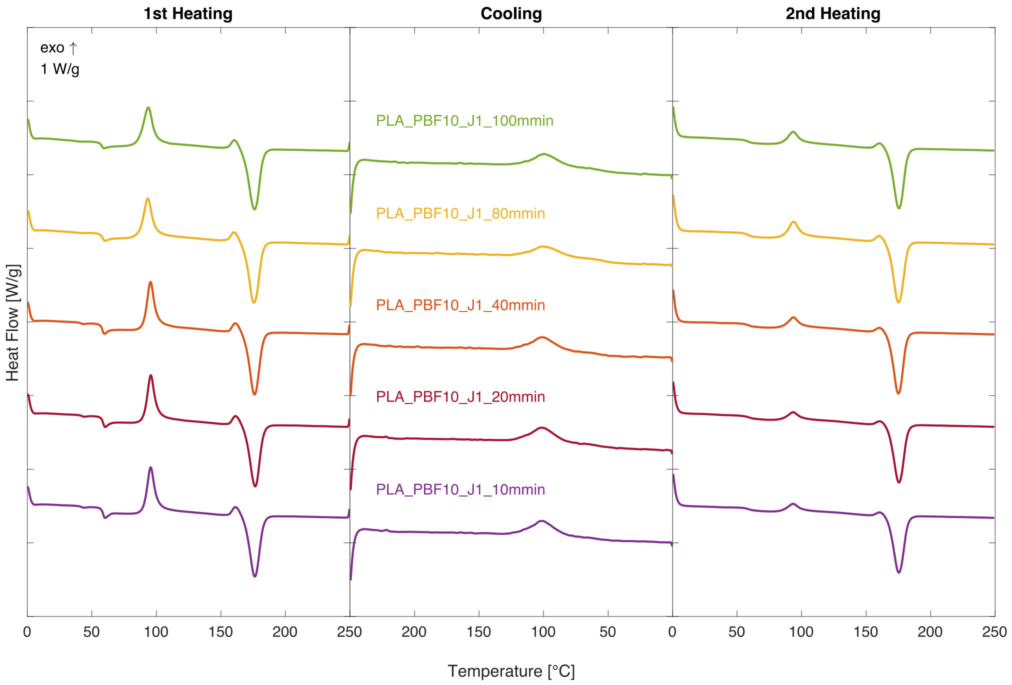

(**e**)

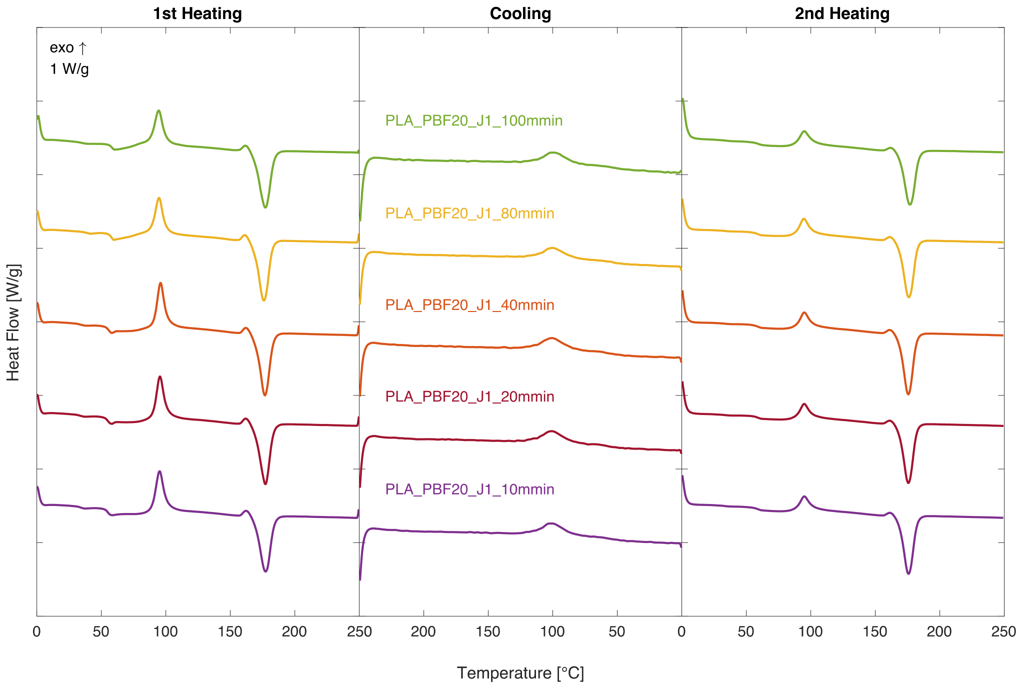

(**f**)

Figure S3. DSC thermograms of the prepared PLA and PLA/PBF fibers collected at different take up speed. (**a**) neat PLA fibers; (**b**) PLA-J1 fibers; (**c**) PLA-PBF1-J1 fibers; (**d**) PLA-PBF-J1 fibers; (**e**) PLA-PBF-J1 fibers; (**f**) PLA-PBF20-J1 fibers.

Table S1. Main results of DSC tests on PLA-Neat fiber samples.

Scan	Property	10 m/min	20 m/min	40 m/min	80 m/min	100 m/min
H1	T_g [°C]	56.4	57.7	57.7	57.4	57.5
	T_{cc} [°C]	91.5	92.2	92.4	92.4	92.6
	ΔH_{cc} [J/g]	28.9	31.3	35.5	33.3	30.5
	T_m [°C]	177.3	177.1	177.3	177.8	177.4

	ΔH_m [J/g]	56.9	59.2	64.4	57.6	55.8
	X_c [%]	29.9	29.7	30.9	26.0	27.0
C	T_c [°C]	99.9	99.6	100.1	99.9	99.5
	ΔH_c [J/g]	36.7	34.6	41.6	36.5	34.1
H2	T_g [°C]	60.5	60.2	60.6	61.2	62.3
	T_{cc} [°C]	/	/	/	/	/
	ΔH_{cc} [J/g]	/	/	/	/	/
	T_m [°C]	176.1	176.2	175.9	176.3	176.3
	ΔH_m [J/g]	55.1	57.3	62.0	55.6	53.6
	X_c [%]	58.8	61.1	66.1	59.4	57.2

H1 = first heating scan, C = cooling scan, H2 = second heating scan, T_g = glass transition temperature, T_{cc} = cold crystallization temperature, ΔH_{cc} = cold crystallization enthalpy, T_m = melting temperature, ΔH_m = melting enthalpy, T_c = crystallization temperature, ΔH_c = crystallization enthalpy, and X_c = crystallization degree.

Table S2. Main results of DSC tests on PLA-J1 fiber samples.

Scan	Property	10 m/min	40 m/min	80 m/min
H1	T_g [°C]	54.8	55.9	55.6
	T_{cc} [°C]	84.8	83.7	82.8
	ΔH_{cc} [J/g]	27.7	26.3	23.4
	T_m [°C]	177.0	174.7	174.3
	ΔH_m [J/g]	49.3	53.1	48.5
	X_c [%]	23.0	28.6	26.9
C	T_c [°C]	99.0	111.9	111.4
	ΔH_c [J/g]	28.1	39.0	35.4
H2	T_g [°C]	59.5	60.4	59.7
	T_{cc} [°C]	92.9	/	/
	ΔH_{cc} [J/g]	6.2	/	/
	T_m [°C]	174.1	174.5	173.9
	ΔH_m [J/g]	42.4	42.5	39.2
	X_c [%]	38.6	45.4	41.9

Table S3. Main results of DSC tests on PLA-PBF1-J1 fiber samples.

Scan	Property	10 m/min	20 m/min	40 m/min	80 m/min	100 m/min
H1	T_g [°C]	56.0	57.5	59.9	60.7	59.9
	T_{cc} [°C]	89.1	87.5	84.7	84.4	84.7
	ΔH_{cc} [J/g]	26.1	23.4	29.5	20.2	19.9
	T_m [°C]	176.3	175.7	175.0	176.9	176.7
	ΔH_m [J/g]	51.6	50.2	69.1	44.9	44.2
	X_c [%]	/	/	/	/	/
C	T_c [°C]	117.3	115.2	112.0	111.5	102.3
	ΔH_c [J/g]	40.8	35.8	41.5	30.0	26.0
H2	T_g [°C]	61.7	59.6	60.0	61.1	60.9
	T_{cc} [°C]	/	93.3	93.5	94.1	94.6
	ΔH_{cc} [J/g]	/	0.8	3.8	1.4	3.9
	T_m [°C]	175.1	175.3	175.7	176.1	176.3
	ΔH_m [J/g]	51.2	40.7	57.4	35.7	36.1
	X_c [%]	/	/	/	/	/

Table S4. Main results of DSC tests on PLA-PBF5-J1 fiber samples.

Scan	Property	10 m/min	20 m/min	40 m/min	80 m/min	100 m/min
H1	T_g [°C]	58.1	56.6	57.1	57.2	57.8
	T_{cc} [°C]	92.9	90.1	87.8	83.5	81.3
	ΔH_{cc} [J/g]	29.6	28.9	28.0	23.1	21.3
	T_m [°C]	176.7	175.8	175.4	174.4	174.3
	ΔH_m [J/g]	48.4	50.6	50.3	48.8	49.2
	X_c [%]	/	/	/	/	/
C	T_c [°C]	102.3	102.6	101.6	101.7	100.8
	ΔH_c [J/g]	29.5	29.7	26.6	29.3	26.4
H2	T_g [°C]	59.4	60.2	60.2	59.6	59.3
	T_{cc} [°C]	92.6	92.7	93.2	92.8	93.0
	ΔH_{cc} [J/g]	3.6	2.7	2.7	2.8	4.5
	T_m [°C]	175.2	175.5	175.7	175.9	175.5
	ΔH_m [J/g]	42.8	43.4	43.1	42.2	42.7
	X_c [%]	/	/	/	/	/

Table S5. Main results of DSC tests on PLA-PBF10-J1 fiber samples.

Scan	Property	10 m/min	20 m/min	40 m/min	80 m/min	100 m/min
H1	T_{gPBF} [°C]	42.3	42.3	41.0	36.4	36.8
	T_{gPLA} [°C]	58.8	58.8	58.2	58.5	57.9
	T_{cc} [°C]	95.6	95.5	95.4	93.3	93.7
	ΔH_{cc} [J/g]	29.4	29.1	29.6	29.7	29.4
	T_m [°C]	176.3	176.6	176.0	175.7	176.0
	ΔH_m [J/g]	46.0	46.8	47.2	48.2	49.4
	X_c [%]	/	/	/	/	/
C	T_c [°C]	101.4	101.1	101.5	100.7	99.32
	ΔH_c [J/g]	23.9	24.3	22.9	20.0	20.6
H2	T_g [°C]	58.7	58.8	58.8	58.7	58.4
	T_{cc} [°C]	93.5	93.7	93.8	93.7	93.6
	ΔH_{cc} [J/g]	5.3	5.7	7.8	12.4	10.2
	T_m [°C]	175.2	175.3	175.0	175.2	175.4
	ΔH_m [J/g]	45.0	44.7	45.6	47.7	46.3
	X_c [%]	/	/	/	/	/

Table S6. Main results of DSC tests on PLA-PBF20-J1 fiber samples.

Scan	Property	10 m/min	20 m/min	40 m/min	80 m/min	100 m/min
H1	T_{gPBF} [°C]	33.0	33.1	33.4	40.1	35.1
	T_{gPLA} [°C]	55.1	55.2	55.8	58.1	58.2
	T_{cc} [°C]	95.2	95.5	95.9	94.7	94.5
	ΔH_{cc} [J/g]	28.8	32.2	33.0	30.1	27.9
	T_m [°C]	177.3	177.1	176.8	176.0	177.2
	ΔH_m [J/g]	45.1	46.0	47.0	46.6	47.8
	X_c [%]	/	/	/	/	/
C	T_c [°C]	100.1	100.3	100.4	100.6	98.5
	ΔH_c [J/g]	19.4	19.2	18.4	16.3	18.7
H2	T_g [°C]	33.6	34.1	33.1	34	34.5
	T_{cc} [°C]	58.3	58.6	58.7	58.3	59.4
	ΔH_{cc} [J/g]	95.0	95.0	94.8	94.7	94.8
	T_m [°C]	12.4	12.4	12.6	14.3	12.2
	ΔH_m [J/g]	175.7	175.7	175.7	176.1	176.9
	X_c [%]	43.7	43.5	43.6	43.9	43.8
	T_{gPBF} [°C]	/	/	/	/	/
